# Modelling population dynamics and seasonal movement to assess and predict the burden of melioidosis

**DOI:** 10.1371/journal.pntd.0007380

**Published:** 2019-05-09

**Authors:** Wiriya Mahikul, Lisa J. White, Kittiyod Poovorawan, Ngamphol Soonthornworasiri, Pataporn Sukontamarn, Phetsavanh Chanthavilay, Graham F. Medley, Wirichada Pan-ngum

**Affiliations:** 1 Department of Tropical Hygiene, Faculty of Tropical Medicine, Mahidol University, Bangkok, Thailand; 2 Mahidol-Oxford Tropical Medicine Research Unit, Faculty of Tropical Medicine, Mahidol University, Bangkok, Thailand; 3 Centre for Tropical Medicine, Nuffield Department of Medicine, University of Oxford, Oxford, United Kingdom; 4 Department of Clinical Tropical Medicine, Faculty of Tropical Medicine, Mahidol University, Bangkok, Thailand; 5 College of Population Studies, Chulalongkorn University, Bangkok, Thailand; 6 Institute of Research and Education Development, UHS, Vientiane, Lao PDR; 7 Centre for Mathematical Modelling of Infectious Disease & Department of Global Health and Development, London School of Hygiene and Tropical Medicine, London, United Kingdom; University of Texas Medical Branch, UNITED STATES

## Abstract

**Background:**

Melioidosis is an infectious disease that is transmitted mainly through contact with contaminated soil or water, and exhibits marked seasonality in most settings, including Southeast Asia. In this study, we used mathematical modelling to examine the impacts of such demographic changes on melioidosis incidence, and to predict the disease burden in a developing country such as Thailand.

**Methodology/Principal findings:**

A melioidosis infection model was constructed which included demographic data, diabetes mellitus (DM) prevalence, and melioidosis disease processes. The model was fitted to reported melioidosis incidence in Thailand by age, sex, and geographical area, between 2008 and 2015, using a Bayesian Markov Chain Monte Carlo (MCMC) approach. The model was then used to predict the disease burden and future trends of melioidosis incidence in Thailand. Our model predicted two-fold higher incidence rates of melioidosis compared with national surveillance data from 2015. The estimated incidence rates among males were two-fold greater than those in females. Furthermore, the melioidosis incidence rates in the Northeast region population, and among the transient population, were more than double compared to the non-Northeast region population. The highest incidence rates occurred in males aged 45–59 years old for all regions. The average incidence rate of melioidosis between 2005 and 2035 was predicted to be 11.42 to 12.78 per 100,000 population per year, with a slightly increasing trend. Overall, it was estimated that about half of all cases of melioidosis were symptomatic. In addition, the model suggested a greater susceptibility to melioidosis in diabetic compared with non-diabetic individuals.

**Conclusions/Significance:**

The increasing trend of melioidosis incidence rates was significantly higher among working-age Northeast and transient populations, males aged ≥45 years old, and diabetic individuals. Targeted intervention strategies, such as health education and awareness raising initiatives, should be implemented on high-risk groups, such as those living in the Northeast region, and the seasonally transient population.

## Author summary

Melioidosis is an infectious disease caused by the Gram-negative bacillus *Burkholderia pseudomallei*, which exhibits marked seasonality in most settings where it occurs, such as Southeast Asia and Northern Australia. Most of the population at risk of contracting melioidosis lives in rural areas; particularly at risk are those who are exposed to soil and water, such as rice farmers. Thailand’s demography is in a transient phase, with older age groups set to double within a decade. Social impacts of lifestyle changes are reflected in seasonal movement and increasing urbanization. In this study, we used mathematical modelling to examine the impacts of such demographical changes on an important infectious disease and to dynamically predict the disease burden in a developing country setting, namely Thailand. We found that melioidosis incidence was significantly higher among working-age Northeast and transient populations, specifically among males aged ≥45 years old and individuals with diabetes. Improved health education and awareness raising should be implemented on a national scale, with a focus on high-risk groups living in endemic areas, as well as those who move seasonally between these and other areas.

## Introduction

Melioidosis is an infection caused by the Gram-negative bacillus *Burkholderia pseudomallei*, which exhibits marked seasonality in most settings where it is endemic, including Southeast Asia and Northern Australia [1]. Melioidosis is a communicable disease that is usually transmitted via contaminated soil or water, and is highly prevalent in Northeast Thailand [2]. Most of the population at risk of melioidosis lives in rural areas, especially those people who frequently come into contact with soil or water, such as rice farmers [3, 4]. In Thailand, the highest number of melioidosis reported cases are often in January and October [5]. Infection with *B*. *pseudomallei* shows great clinical diversity, spanning asymptomatic infections, localized skin ulcers or abscesses, chronic pneumonia mimicking tuberculosis, and fulminant septic shock with abscesses in multiple internal organs [6]. Both humans and animals are susceptible to *B*. *pseudomallei*, and may be infected by percutaneous inoculation, inhalation, or ingestion. Person-to-person spread and zoonotic infections of humans are very rare [7]. The incubation period is between 1–21 days (average 9 days) [8], and is believed to be influenced by the inoculation dose, mode of infection, host risk factors, and probably differential virulence of the infecting organisms. Most cases result from recent infections, although latency with reactivation has been described up to 62 years following exposure [8], while the median times to relapse and reinfection are 21 weeks and 111 weeks, respectively. The risk of relapse is related to a patient’s adherence to treatment and the initial extent of disease, but not to any underlying conditions [9–11]. Melioidosis seems to be more severe in older people with lower immunity or chronic underlying conditions, such as diabetes [12]. The risk of contracting melioidosis in diabetic individuals is 12 times higher than for non-diabetic individuals [13, 14]. Currently, the global burden of melioidosis is estimated to be 165,000 cases per year (95% credible interval 68,000–412,000), with 89,000 deaths (36,000–227,000) [15].

Thailand’s Bureau of Epidemiology (BoE) launched a melioidosis surveillance system in 2001 (Report 506) [5]. Approximately 80% of reported melioidosis cases were from Northeast Thailand [5]. In the past, the number of cases shown in the surveillance system was heavily relied on provincial and regional hospitals voluntarily report, very few were reported from private hospitals [16]. In general, melioidosis is diagnosed by testing for antibodies to *B*. *pseudomallei* using an indirect hemagglutination (IHA) technique, which has been found to have low sensitivity and specificity [17]. This surveillance system was revised in 2010 in order to capture more health data items. There has been an increase in usage of bacterial culture [16] which could give rise to an increase in total number of culture-confirmed cases. In addition, there has been an improvement to access to healthcare. Nevertheless, the true number of cases is still under-reported because of diverse clinical manifestations and inadequate bacterial identification methods. A previous estimation suggested cases in Thailand were in excess of 7,000 cases per year [15], while the BoE reported just 3,242 cases in 2015 [5].

*B*. *pseudomallei* is resistant to a wide range of antimicrobials, and ineffective treatment may result in death in 70% of cases [18]. The treatment for melioidosis consists of an intensive phase of at least 10–14 days of ceftazidime, meropenem, or imipenem, administered intravenously, followed by oral eradication therapy, usually with trimethoprim–sulfamethoxazole (TMP-SMX) for 3–6 months [19]. There is currently no vaccine against melioidosis [20, 21].

The demographics of Thailand are currently in a transition phase, becoming more like those of developed countries, with rapid changes in population structure, reductions in birth and mortality rates, and a low rate of population growth. Urbanization is accelerating, and there are large annual population movements. These types of changes have been shown to have important impacts on public health and the disease burden of both non-communicable [22] and communicable diseases [23]. The population at highest risk of contracting melioidosis is the working age group. There is appreciable seasonal movement among this group as they go about their working lives. The internal migration of Thai people involves a number of distinct forms of movement within each year. Three forms have been identified in previous research [24]: a single movement, seasonal movement, and repeated movement. Seasonal migration involves people moving from the North and Northeast regions of Thailand towards the Bangkok metropolis and the Central region during the dry season (from March through to June), and in the reverse direction during the wet season (June to September) [24]. 40% of people from the Northeast are classified as seasonal migrants (a transient population) [25]. It is obvious that for person-to-person transmissible infections, there are significantly more infections when such transient individuals are considered [26–29]. However, very few studies were trying to look at the effect of transient populations on an infectious disease from a primarily environmental source which will help better describe the temporal and spatial changes of the incidence of such a disease [30]. Developed countries are also observing an emergence of melioidosis related to travelling and importation of cases [1].

To date, only a few approaches have been applied to determine the melioidosis burden, including simple maps of melioidosis [1], maps of the global distribution of *B*. *pseudomallei*, and estimates of the total incidence and mortality due to melioidosis worldwide using a statistical model [15]. Only one study has used mathematical modelling, exploring the use of childhood seroprevalence data as a marker of intensity of exposure [31]. In this study, we used mathematical modelling to predict the incidence of melioidosis in the Thai population, taking account of population changes, seasonal movement, and incidence of diabetes. The model provides multi-dimensional forecasting of melioidosis, which could be useful for targeting intervention strategies in this setting.

## Methods

### Demographic and seasonal movement sub-models

We generated a deterministic demographic sub-model to predict the size of the total population (see S1 Figure A). We stratified the population by age and sex into 100 annual interval classes, from 0 to 100 years old. The population in each class followed the actual population structure of Thailand between 1980 and 2015, based on birth, death, and migration rate data from the Population and Housing Census [32, 33], and using the 1980 census data as the initial condition. All females in the age classes between 15 to 50 years old were considered to be capable of reproduction, with the fertility rate (*fr*) [34], while the death rate was age-related [35]. Members of the population were assumed to die upon reaching 100 years of age. Crude net migration rates (immigrant minus emigrant per 1,000 population) for each year had an impact on all age and sex compartments [36]. Most of the at-risk population for melioidosis lives in rural areas, especially in Northeast Thailand, so we modelled internal migration by classifying the population of Thailand into three independent groups. These were: those from the Northeast region who live at home for more than 6 months in a year (*NE*), the transient group or the people from the Northeast region who move seasonally between home and other parts of the country and spend less than 6 months in a year at home (*T*) and lastly the non-Northeast group, who live somewhere other than the Northeast (*Non*_*NE*). We created the seasonal movement sub-model to overlay with the demographic sub-model to estimate the rates of movement among them (see S1 Figure B). We solved a large set of ordinary differential equations (ODE) for the deterministic demographic sub-model and the seasonal movement sub-model, defined in S1 Information on Demographic sub-model and Seasonal movement sub-model, respectively.

### Melioidosis infection model

**Fig 1:**
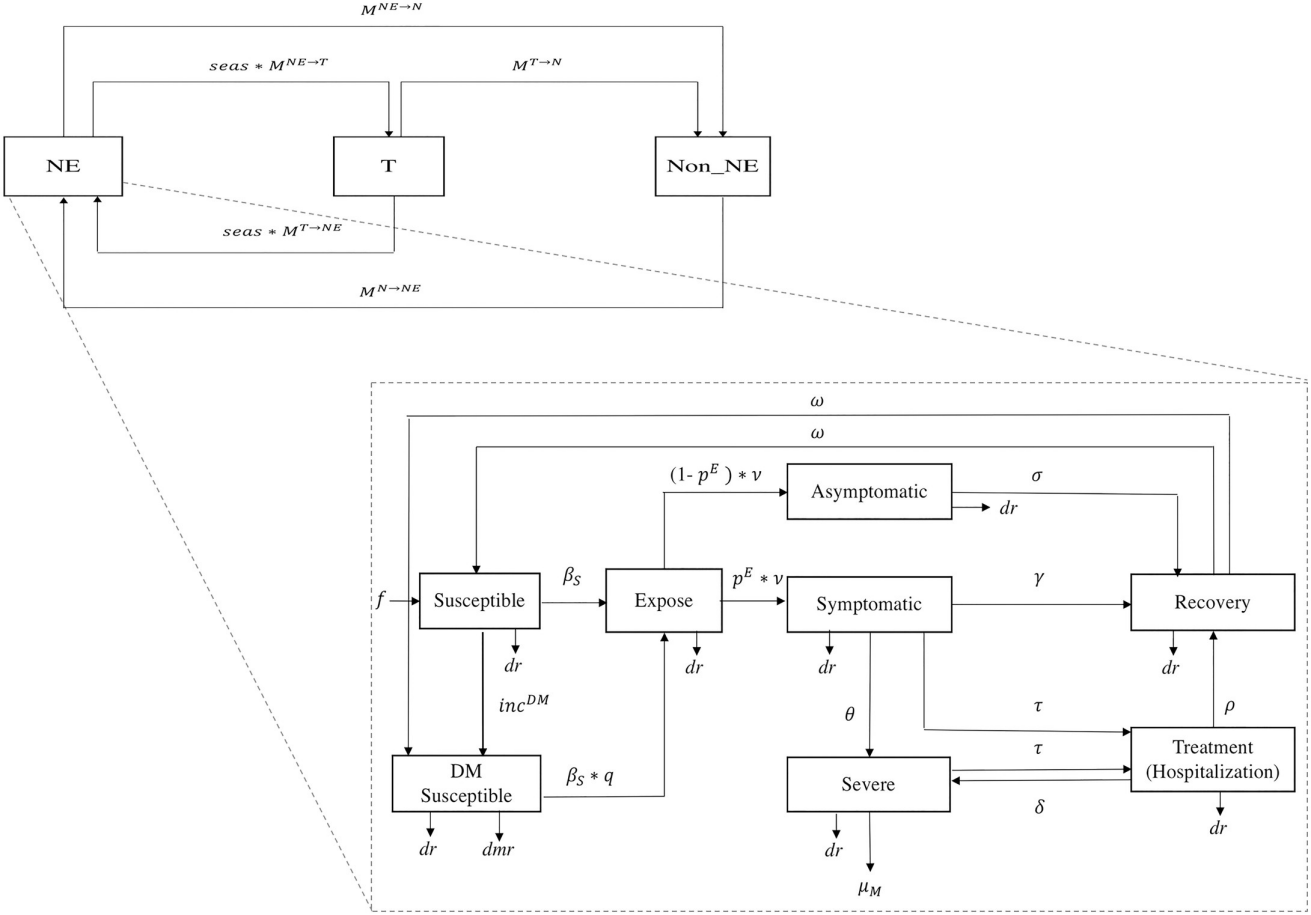
Schematic representation of the melioidosis dynamic sub-model (SEIRS model), with the population further divided into eight health compartments with 100 age categories: Susceptible, diabetic susceptible, exposed, symptomatic, asymptomatic, severe, treatment, and recovery.

The demographic and seasonal movement sub-model was overlaid with the melioidosis infection model, defined in S1 Information on Melioidosis infection sub-model. In the melioidosis infection model (a susceptible, exposed, infected, recovered, susceptible, or SEIRS, model), the population was further divided into eight health compartments: susceptible (S), diabetic susceptible (SDM), exposed (E), symptomatic (Sym), asymptomatic (Asym), severe (Sev), treatment (Treat), and recovered (R) (see Fig 1).

Melioidosis case data stratified by age, sex, and geographical area were obtained from the Thai annual epidemiological surveillance reports from 2008 to 2015 [5]. Key assumptions for our model were as follows. First, the transient population data used within this model referred only to the movement of the Thai population. The movement of migrant workers from other countries could be significant but was omitted in this study for simplicity [24]. Second, diabetes progression was assumed to be irreversible, i.e. people could not move from diabetic to non-diabetic. Third, we did not consider pre-diabetes or impaired glucose tolerance. Fourth, we assumed that incidence rates of diabetes were constant over time but varied by age. Fifth, we did not focus on chronic symptoms (those of duration greater than two months), including such presentations as chronic skin infections, chronic lung nodules, or pneumonia, which only accounted for around 10% of melioidosis patients [12]. Finally, we did not focus on any behavioral factors such as excessive alcohol use.

We used R software version 3.3.3 to run and analyze the model outputs, and the deSolve package to solve the differential equations [37]. The initial parameter values were calculated from population data and disease burden. Model fitting was carried out using the Markov Chain Monte Carlo (MCMC) method, implemented with the Bayesian Tools R package as defined in S2 Information on the Bayesian framework [38]. The demographic and seasonal movement sub-models were run from 1980 (see S2 Figure A) to calibrate the model by fitting to the average migration data, including the population in the Northeast moving to non-Northeast, and the reverse direction from 2005 to 2015 [25]. We estimated seasonal movement parameters from the transient population model (see S1 Table A) and used them to run the melioidosis infection model from 2005. The model was run and fitted to the annual incidence of melioidosis by age, sex, and area by year, and seasonally by month, from 2008 to 2015 [5]. For model fitting, the DEzs method in the Bayesian Tools package allowed automatic parallelization on three cores to be used for sampling. This method allowed fewer chains to be used for estimated a large number of parameters and thus optimized the computational time [39]. Number of iterations and burn in were decided upon the model convergence by analyzing the differences between multiple Markov chains. The convergence was assessed by several measures including the standard procedure of Gelmal-Rubin [40, 41] and the target acceptance rates [42]. Thirty-three parameters were estimated and the median values and credibility intervals were reported. These parameters were those representing the infection rates among both sexes in the Northeast, transient, and non-Northeast populations, ($\beta_{a}^{NE}$,$\;\beta_{a}^{T}$,$\;\beta_{a}^{N}$) respectively, proportion of symptomatic cases (*p*^*E*^), recovery rate from asymptomatic (σ), recovery rate from symptomatic (γ), Relative susceptibility to melioidosis among diabetic individuals when compare with non-diabetic (*q*), mortality/death rate for melioidosis (*μ*_*M*_), amplitude (*A*_*inc*_), phase angle (*φ*_*inc*_) and proportion of reporting (*Report*) (see S1 Table A). Note that the proportion *(1- Report)* was defined as “Under-reporting” i.e. those symptomatic melioidosis patients that have been seen by a physician, but the physician did not report them to the public health authority for some reasons e.g. improperly diagnosis or missing report. The model was further used to predict the 20-year age-specific incidence of melioidosis among males and females in Thailand, sampling all 33 parameters from the posterior chains. The model predictions were reported as age, gender, and area-specific incidence rates over time.

## Results

The demographic sub-model was able to reproduce the past population structure of Thailand from 1980 to the present (see S2 Figure A). The parameters that characterized seasonal movement were estimated by fitting the model to the population movement data (see S2 Figure B). The model showed that majority of movements were made by Northeast individuals who moved to non-Northeast areas, approximately 13,600 persons per 100,000 population per month, or 34% of all movements within a month (see S1 Table A). Moreover, the majority of movements were among those aged between 15 and 60 years old, about 19,000 persons per 100,000 population per month, or 51% of all movements within a month (see S2 Figure C).

**Fig 2:**
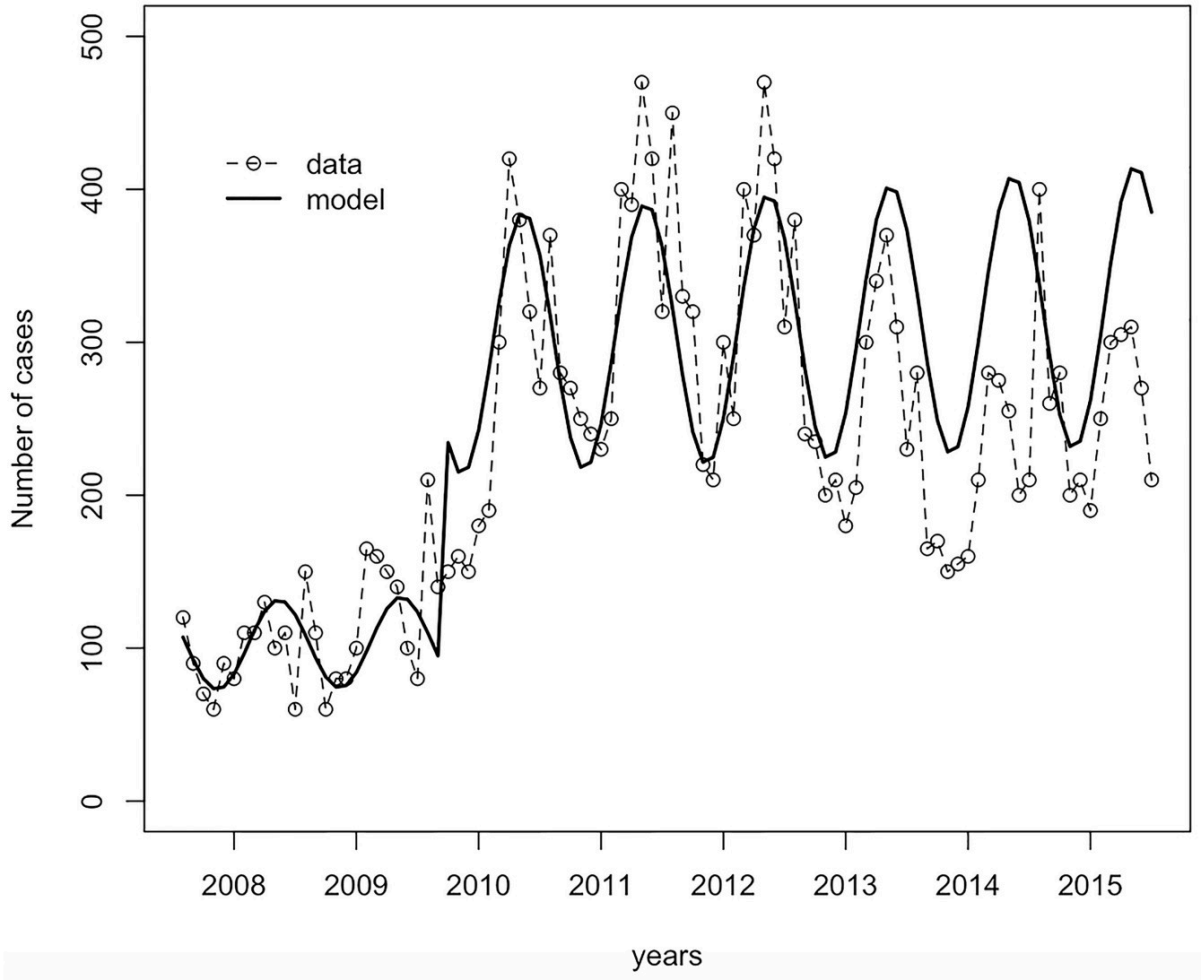
Comparing the observed and model estimates of monthly melioidosis cases between 2005 and 2015.

The fitting performance is shown in Fig 2. Melioidosis cases occurred seasonally, with a peak in the wet season that lasted from May to October. The infection parameters that minimized the fit statistic, using the Bayesian method, are shown in Table 1. The highest infection rate was estimated to be 6 cases per 100,000 population per month among males aged 45–59 years old in the Northeast. The lowest rate was 0.4 cases per 100,000 population per month among females aged 15–44 years old in the non-Northeast region. Surprisingly, we found that the infection rate among the transient male population aged 15–44 years was higher than the non-Northeast population (0.8 compared with 0.08 per 100,000 persons per month). Overall 46% of melioidosis cases were symptomatic. Recovery rates for untreated symptomatic cases and asymptomatic patients were estimated by the model, with the average period of infection estimated at around 9 and 5 months, respectively. The susceptibility to melioidosis among DM population is 10.84 [95% CI 8.42–12.23] times greater than the non-DM population. If patients’ treatment failed and they developed severe melioidosis, they could die within two weeks. We estimated 80% and 50% under-reporting of cases in 2008–2009 and 2010–2015, respectively.

**Table 1:** Results of estimated parameters of the melioidosis model.

Parameter	Symbol	Value (95% Credible Interval)
**Infection rate (10^−5^) among males in the Northeast (per capita per month)**	$\beta_{ma}^{NE}$	Aged 0–14 = 0.7 (0.6–0.8) Aged 15–44 = 0.7 (0.5–0.8) Aged 45–59 = 6.1 (4.2–7.3) Aged > = 60 = 1.7 (1.3–1.8)
**Infection rate (10^−5^) among females in the Northeast (per capita per month)**	$\beta_{fa}^{NE}$	Aged 0–14 = 0.5 (0.4–0.6) Aged 15–44 = 0.3 (0.2–0.4) Aged 45–59 = 2.8 (1.1–2.9) Aged > = 60 = 0.8 (0.6–0.9)
**Infection rate (10^−5^) among males in the transient population (per capita per month)**	$\beta_{ma}^{T}$	Aged 0–14 = 0.3 (0.1–0.5) Aged 15–44 = 0.8 (0.5–1.2) Aged 45–59 = 0.8 (0.2–1.2) Aged > = 60 = 1.1 (0.2–1.3)
**Infection rate (10^−5^) among females in the transient population (per capita per month)**	$\beta_{fa}^{T}$	Aged 0–14 = 1.6 (0.1–1.8) Aged 15–44 = 0.3 (0.1–1.1) Aged 45–59 = 0.3 (0.1–0.6) Aged > = 60 = 0.7 (0.2–1.2)
**Infection rate (10^−5^) among males in the non-Northeast (per capita per month)**	$\beta_{ma}^{N}$	Aged 0–14 = 0.06 (0.05–0.08) Aged 15–44 = 0.08 (0.04–0.09) Aged 45–59 = 0.47 (0.4–0.65) Aged > = 60 = 0.13 (0.12–0.2)
**Infection rate (10^−5^) among females in the non-Northeast (per capita per month)**	$\beta_{fa}^{N}$	Aged 0–14 = 0.09 (0.03–0.17) Aged 15–44 = 0.04 (0.02–0.05) Aged 45–59 = 0.19 (0.15–0.3) Aged > = 60 = 0.08 (0.04–0.09)
**Proportion of symptomatic cases from exposer**	*p*^*E*^	0.46 (0.38–0.53)
**Recovery rate from asymptomatic (per capita per month)**	*σ*	0.21 (0.005–0.24)
**Recovery rate from untreated symptomatic (per capita per month)**	*γ*	0.11 (0.01–0.15)
**Relative susceptibility to melioidosis among diabetic individuals**	*q*	10.84 (8.42–12.23)
**mortality/death rate for melioidosis (per capita per month)**	*μ*_*M*_	0.6 (0.4–0.75)
**Proportion of reporting**	*Report*	*Report* (in 2005–2009) = 0.17 (0.15–0.22) *Report* (in 2010–2015) = 0.43 (0.39–0.54)
**Amplitude**	*A*_*inc*_	1.7 (1.3–1.8)
**Phase angle**	*φ*_*inc*_	20.6 (20.3–21.0)

**Fig 3:**
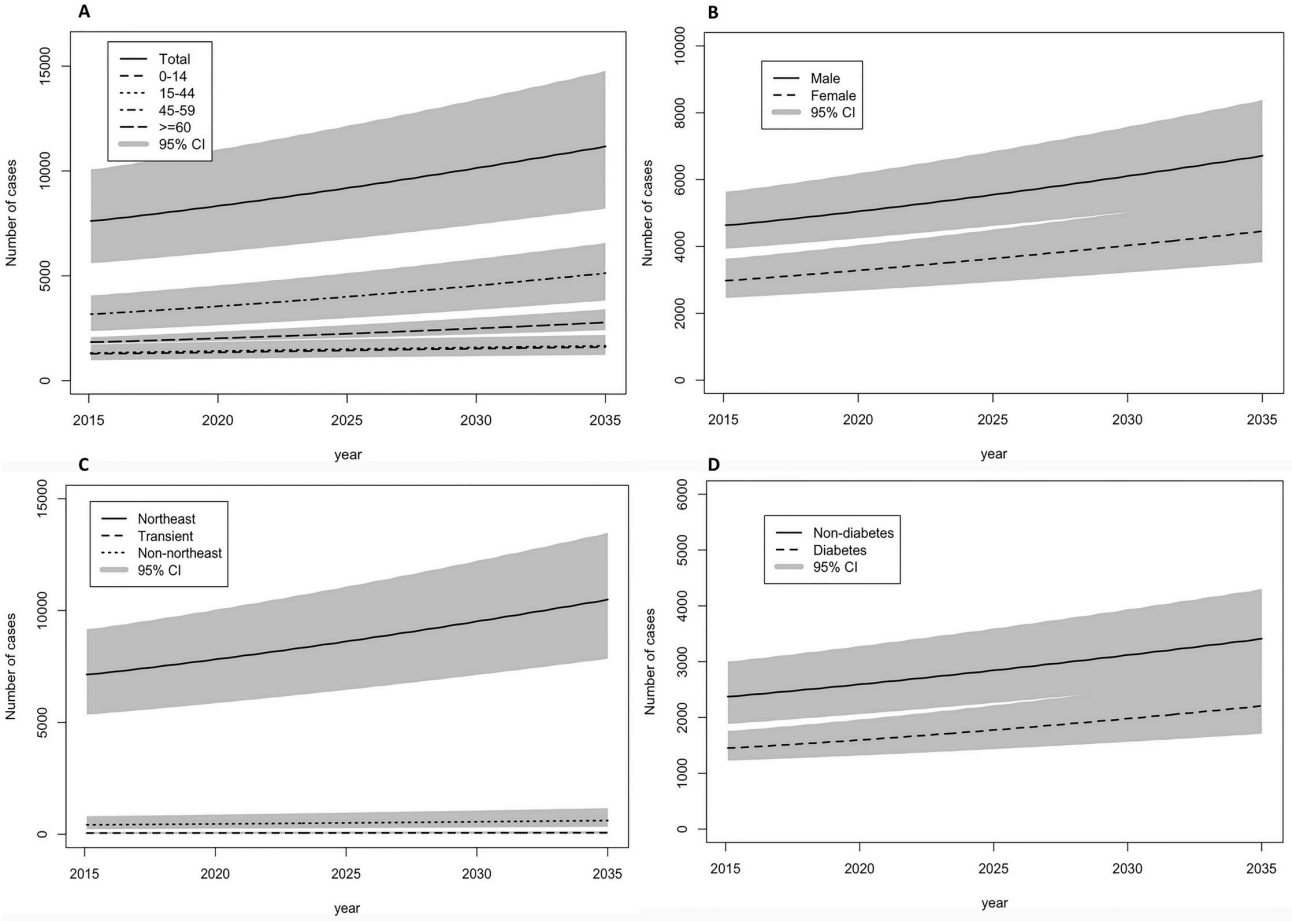
Projections of the numbers of melioidosis cases (95% credible intervals) between 2015 and 2035 in Thailand: (A) By age class (B) By gender (C) By geographical area, and (D) By diabetes status.

Projections of the numbers of melioidosis cases between 2015 and 2035 are given in Fig 3. Total melioidosis incidence per year was projected to increase by 70%, from 6,569 (4,834–8,701) in 2015 to 11,173 (8,207–14,773) in 2035. The largest increase of melioidosis was projected to occur among the population aged 45–59 years old. The predicted incidence among males was two-fold greater than that of females. The majority of melioidosis cases were seen to occur in the population from the Northeast region of Thailand. The predicted incidence among non-diabetic was two-fold greater than that of diabetic population.

**Fig 4:**
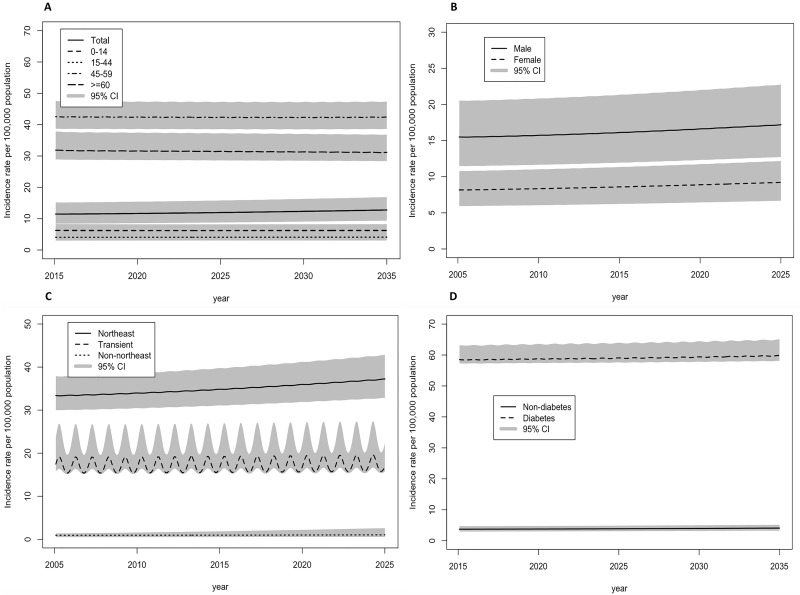
Projections of incidence rate of melioidosis per 100,000 population (95% credible intervals) between 2015 and 2035 in Thailand: (A) By age class (B) By gender (C) By geographical area, and (D) By diabetes status.

In Fig 4, total melioidosis incidence rates were projected to increase by approximately 10% by 2035, from 11.42 (8.5–13.4) in 2015 to 12.78 (9.6–14.9) per 100,000 population in 2035 (see Table 2). The highest incidence rates were predicted to be among those aged between 45–59 years old, followed by those age 60 years old and above. The incidence was almost double among males compared with females in both Northeast and other regions. The incidence rate among the Northeast population was more than double compared with the transient population, and almost ten times higher when compared with the other regions. This study also highlighted the importance of melioidosis among the transient population who temporally live in the risk area but had almost six times higher incidence compared with other regional populations. From diabetes prospective, the incidence of melioidosis among diabetes was predicted to be as high as 60 per 100,000 population. To summary, the risk of melioidosis among the aging population with some chronic diseases such as diabetes is presenting an increasing trend. The risk of infection among transient population, who temporary get some disease exposure during the agricultural seasons, cannot be ignored.

**Table 2:** Projection of melioidosis incidence rates per 100,000 people in males and females by age group for selected years in each area.

Year	Age group (years)								Total
	0–14		15–44		45–59		≥60		
	Male	Female	Male	Female	Male	Female	Male	Female	
**Total**									
**2005**	7.73	4.27	5.82	2.82	**63.97**	**29.82**	43.61	24.82	11.42
**2010**	7.74	4.27	5.82	2.82	**63.79**	**29.78**	43.59	24.77	11.39
**2015**	7.67	4.25	5.83	2.83	**63.66**	**29.75**	43.48	24.65	11.46
**2035**	7.64	4.25	5.86	2.85	**63.58**	**29.74**	42.94	24.59	12.78
**Northeast**									
**2005**	22.43	11.86	16.77	8.18	**185.24**	**86.27**	124.61	70.34	33.24
**2010**	22.44	11.84	16.75	8.18	**184.75**	**86.14**	124.55	70.22	33.20
**2015**	22.24	11.81	16.76	8.19	**184.36**	**86.06**	124.22	69.87	33.40
**2035**	22.16	11.79	16.87	8.25	**184.15**	**86.02**	122.70	69.70	37.22
**Transient**									
**2005**	11.57	32.95	17.49	7.10	**73.46**	30.33	65.85	**52.83**	16.24
**2010**	11.99	34.21	18.32	7.43	**75.71**	31.01	67.19	**54.25**	16.01
**2015**	11.89	34.11	18.34	7.44	**75.37**	30.97	67.01	**53.89**	15.83
**2035**	11.88	34.10	18.48	7.49	**74.08**	30.98	66.95	**53.74**	16.93
**Non-Northeast**									
**2005**	0.62	0.46	0.54	0.27	**4.79**	**2.26**	3.33	2.15	0.93
**2010**	0.62	0.46	0.55	0.26	**4.78**	**2.26**	3.33	2.15	0.94
**2015**	0.61	0.45	0.55	0.26	**4.77**	**2.26**	3.32	2.14	0.94
**2035**	0.61	0.45	0.56	0.26	**4.76**	**2.25**	3.28	2.13	1.04

## Discussion

Few models have been used to predict the incidence of melioidosis on either a national or global scale [14, 15, 43]. We applied population dynamics, seasonal movement, and the impact of diabetes to study melioidosis epidemiology in Thailand. Other approaches such as decision tree or Markov model can also be used to study melioidosis epidemiology given that the rate of transmission is constant and the system is linear. Our model fits a dynamic model for a non-transmissible disease to data on notifications only, which should allow reasonable predictions to be made as to the future course of the epidemic. However, drawing strong inferences regarding parameter values that pertain to transitions through infection/disease states after the point of infection is less safe, such that particular caution should be exercised in regards to parameters, especially those discussed here. Our findings have some similarities and differences compared with previously published work. Limmathurotsakul and colleagues used a negative binomial model to derive estimates of 7,572 (3,396–17,685) cases for global melioidosis incidence in the year 2015 [15]. This figure was similar to the incidence of melioidosis estimated in our study, which was 7,569 cases (4,834–8,701) for 2015. Both studies reached similar estimates of approximately 50% for case reporting and 40–45% for mortality/death rate for melioidosis (see S2 Table A), while the risk factors identified for melioidosis were also in agreement, i.e. being male, aged more than 44 years old, and having diabetes [13]. Two previous studies by both Thai and Australian researchers consistently showed that type 2 diabetes increased the risk of melioidosis by more than 10 times when compared with those non-diabetes [13, 44], this figure is similar to our model estimates. Buckee and colleagues pointed out that seasonal disease incidence could be driven by the mobility and aggregation of human populations, which spark outbreaks and sustain transmission [30]. Northeast and transient males aged more than 45 years old were also predicted by our model to be a highest risk groups for melioidosis. Apart from reporting and mortality/death rates for melioidosis, the model also gave the estimates for some natural history of disease parameters which would be hard to measure i.e. proportion and duration of asymptomatic infections, duration of untreated symptomatic infections, and host susceptibility to melioidosis [45]. With regard to asymptomatic infections, a few studies have tried to characterize and estimate the number of these hidden infections [44–46]. Our model suggested that there could be a significant proportion of asymptomatic infections (54%). Although the parameter values used in the fitting and prediction process are based on annual incidence data only, the variation in the parameter values is included to reflect the uncertainty in the predictions, and the posterior distributions represent sets of collinear parameters that reproduce the observed data. Further studies and more appropriate data are required to refine these parameters.

Our model can provide many benefits for health policy planning. First, the model, in common with previous studies, estimated that only about half of all melioidosis cases were being reported. Under-reporting results in melioidosis being neglected, even more than other neglected diseases such as dengue and leptospirosis [16]. Previous study suggested that melioidosis was the third most frequent cause of death from infectious diseases in northeast Thailand, after HIV/AIDS and tuberculosis [13]. By regarding melioidosis as being less important disease has made it being further under-recognized by healthcare professionals, low health budgets to invest in intensive prevention and control, poor disease knowledge and practices among the population at risk, and finally a lack of research that would enable the development of concrete strategies to improve standards of care. Second, the model can be used to guide the design of targeted interventions i.e. predicting and identifying populations at high risk for morbidity and mortality. In line with the model’s predictions, targeted intervention strategies could be concentrated among the male population of working age who live in the Northeast, as well as the transient population. These strategies could include providing health education to increase protective practices while engaging in agricultural activities, washing after work, and seeking appropriate health advice when feeling sick. To prevent deaths due to infections in older age groups, i.e. 45 years or older (65%) (see S2 Table A), national strategies could focus on early diagnosis and appropriate treatment, as well as improving diabetes screening programmes, since elderly people with diabetes may be prone to severe melioidosis.

Our model has some limitations. For simplicity we assumed that diabetes influenced the likelihood of melioidosis infection alone and therefore once the person is infected with melioidosis, the diagnosis and disease progression are independent of diabetes status. Although diabetes has been shown to play roles in increasing severity and/or that medication of diabetes may also affect susceptibility and presentation of melioidosis [47, 48]. We also assumed that mortality/death rates for melioidosis, incidence rates of diabetes, and seasonal movement rates were constant over time, although they varied by age. It has been suggested that mortality/death rate for melioidosis due to diabetes have decreased over time because of improved access to hospitals [49], and lifestyle changes might also have affected incidence rates [50]. In this model we classified the population into those living in the Northeast and non-Northeast, which meant that the model was unable to predict the incidence of melioidosis in locations more specifically than non-Northeast. It is important to keep in mind that melioidosis is probably prevalent in all regions of Thailand, the lack of knowledge, disease awareness and diagnosis tools led to heavily report of cases by the Northeast region only [16]. We assumed that the estimates of reporting among both males and females were the same. The annual epidemiological surveillance reports of melioidosis data used in the model included cases from all provinces around Thailand, except for those cases seen in private hospitals, which account for about 30% of hospital provision, although there is no information on the relative likelihood of melioidosis being diagnosed in different sectors. Melioidosis diagnoses reported annually by the BoE are made using an indirect hemagglutination (IHA) technique to test for antibodies to *B*. *pseudomallei*, which has been found to have low sensitivity and specificity. It could therefore potentially under-predict the number of cases.

### Conclusion

Population dynamics, seasonal movement, melioidosis infection rates, and under-reporting are important components of melioidosis incidence patterns. The increases seen in melioidosis cases are partly attributable to demographic changes as working, transient, and aging population groups are more prone to develop melioidosis. The key findings from our study are firstly, the increasing trend of melioidosis incidence, especially among males aged 45–59 years old, is predicted to continue; and secondly, the male, Northeast, and transient populations aged 45–59 years old were at the highest risk of melioidosis infection.

We anticipate that the modelling methods described here could be used in similar settings, especially those with reliable census data, to estimate the future melioidosis burden, as well as the potential effects of under-reporting. In addition, this modelling approach could be adapted to study other infectious diseases, behavioral changes, and seasonal movements, where demographic factors are important drivers of a population’s disease burden.

## Supporting information

S1 File Figure **(A)**. Schematic representation of the deterministic demographic model. Figure **(B).** Schematic representation of the seasonal movement sub-model. Table **(A).** Parameter table for melioidosis infection model. **Demographic sub-model.** Set of ordinary differential equations (ODE) for the demographic sub-model. **Seasonal movement sub-model.** Set of ordinary differential equations (ODE) for the seasonal movement sub-model. **Melioidosis infection sub-model.** Set of ordinary differential equations (ODE) for the melioidosis dynamics sub-model.
(DOCX)

S2 File Figure **(A).** Projection of the population size of Thailand between 1980 and 2035. Figure **(B).** Observed data and model estimates of the annual population (in millions) in Northeast and non-Northeast Thailand during 2005–2010. Figure **(C)**. Estimation of the transient population by age (in thousands) in Thailand, from 2005 to 2015. Figure **(D)**. Posterior distributions from the melioidosis infection model, that each row corresponds to the separate parameter, the left-hand column contains traces with 6 color chains and the right-hand column contains the posterior distribution, corresponding to each parameter.Table **(A)**. Estimation of the number of deaths of males and females from melioidosis by age group for selected years. **Bayesian framework**. Bayes theorem, Prior distribution, Likelihood function and Posterior estimation.
(DOCX)
