# Supplementary material for: Modelling population dynamics and seasonal movement to assess and predict the burden of melioidosis
Source: PLoS Negl Trop Dis. 2019 May 9;13(5):e0007380. doi: 10.1371/journal.pntd.0007380 (PMC6529009; doi:10.1371/journal.pntd.0007380)
Supplement: S1 File — Figure (A). Schematic representation of the deterministic demographic model. Figure (B). Schematic representation of the seasonal movement sub-model. Table (A). Parameter table for melioidosis infection model. Demographic sub-model. Set of ordinary differential equations (ODE) for the demographic sub-model. Seasonal movement sub-model. Set of ordinary differential equations (ODE) for the seasonal movement sub-model. Melioidosis infection sub-model. Set of ordinary differential equations (ODE) for the melioidosis dynamics sub-model. (DOCX) [file pntd.0007380.s001.docx]

Supporting Information 1 (S1)


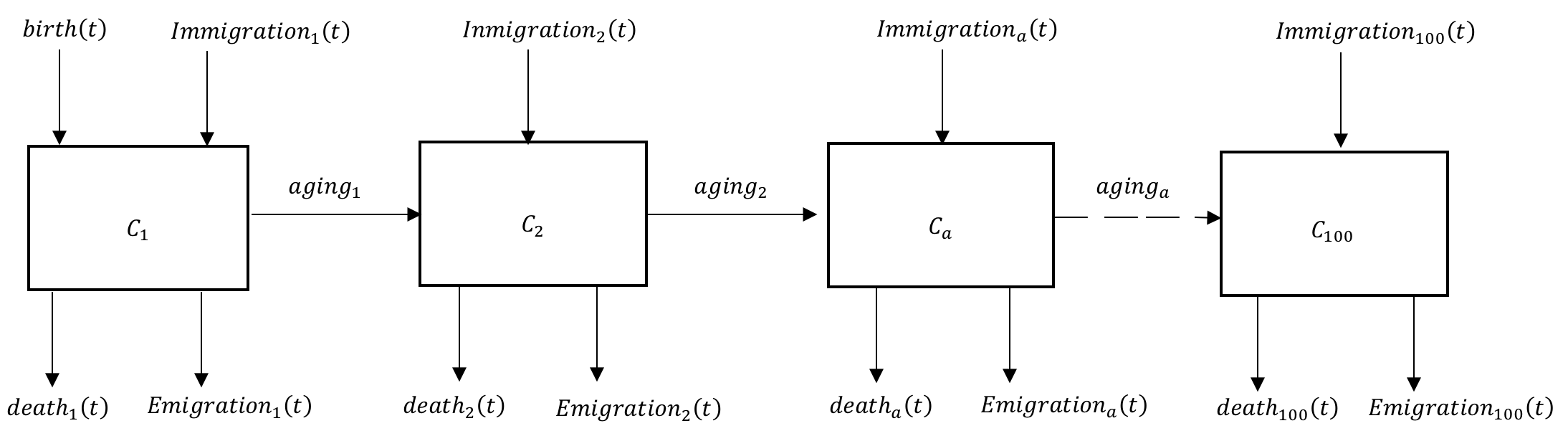


## **Figure A. Schematic representation of the deterministic demographic** **model.**

# Demographic sub-model

We solved a large set of ordinary differential equations (ODEs) for the deterministic demographic sub-model (Figure A). Let $C_{a}(t)$ be the number of people of at age, *a*, at time, *t* and ${fr}_{a}$ be the fertility rate in female aged *a* years old [1]. The number of newborn babies at any time *t* is shown as follows:

$birth\left( t \right)=\sum_{a} {fr}_{a}{.C}_{a} \left( t \right)$ (Equation 1)

Deaths [2] among males and females were calculated from the age-specific mortality rate, *dr_a_:*

${death}_{a}\left( t \right)={dr}_{a}.C_{a}(t)$ (Equation 2)

Net migration (immigration minus emigration) [3] among males and females was calculated from the migration rate, *mr_a_:*

${migration}_{a}\left( t \right)={mr}_{a}.C_{a}(t)$ (Equation 3)

*Aging* is the monthly rate at which individuals move to the next age group, represented as $\frac{1}{age.diff}$*,* where $age.diff$ represented the difference between two age classes. In this model the $age.diff$ is always equal to one year. We modelled the population dynamics by 1-year age classes using the ODEs and generated the matrix equation for individual dynamics, which describes the balance between birth inflows, aging, migration, and death, as follows:

$\left[ \begin{matrix} \frac{{dC}_{1}}{dt} \\ \frac{{dC}_{2}}{dt} \\ \frac{{dC}_{3}}{dt} \\ \vdots\\ \frac{{dC}_{100}}{dt} \end{matrix} \right]=\left[ \begin{aligned} birth(t) \\ 0 \\ 0 \\ 0 \\ 0 \end{aligned} \right]+\left[ \begin{matrix} {migration}_{1}(t) \\ {migration}_{2}(t) \\ {migration}_{3}(t) \\ \vdots\\ {migration}_{100}(t) \end{matrix} \right]+\left[ \begin{matrix} -{aging}_{1} & 0 & 0 & \cdots& 0 \\ {aging}_{1} & -{aging}_{2} & 0 & \cdots& 0 \\ 0 & {aging}_{2} & -{aging}_{3} & \cdots& 0 \\ \vdots& \vdots& \vdots& \ddots& \vdots\\ 0 & \cdots& \cdots& {aging}_{99} & {-aging}_{100} \end{matrix} \right]\left[ \begin{matrix} C_{1} \\ C_{2} \\ C_{3} \\ \vdots\\ C_{100} \end{matrix} \right]-\left[ \begin{matrix} {death}_{1}(t) \\ {death}_{2}(t) \\ {death}_{3}(t) \\ \vdots\\ {death}_{100}(t) \end{matrix} \right]$
 (Equation 4)


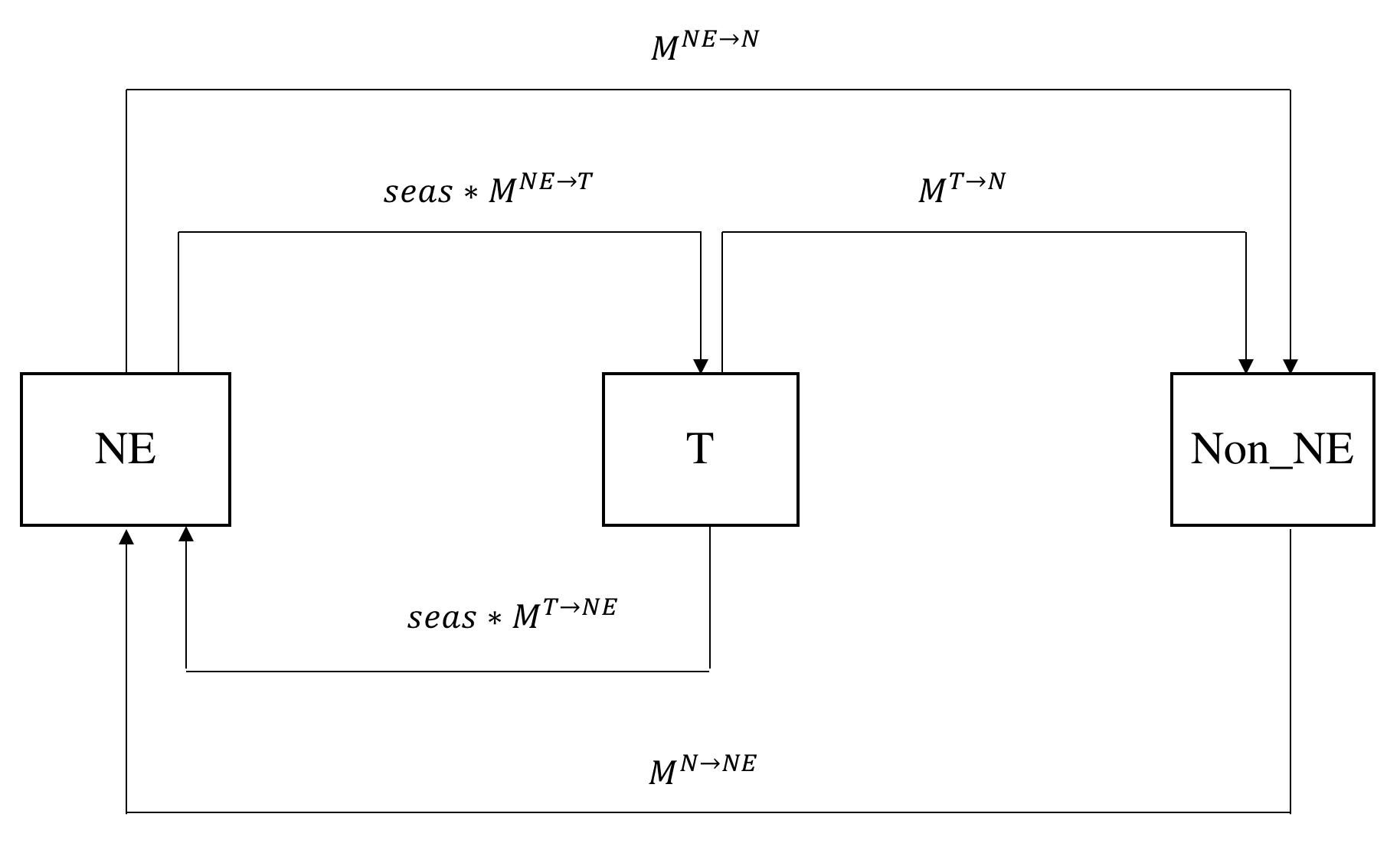


Figure B. Schematic representation of the seasonal movement sub-model.

# Seasonal movement sub-model

We solved a large set of ordinary differential equations (ODEs) for the seasonal movement sub-model. Let ${NE}_{a}(t)$, $T_{a}(t)$, ${Non\_NE}_{a}(t)$ be the number of Northeast, transient, and non-Northeast people at age, *a*, at time, *t*.

Seasonal movement ($Seas(t)$) are sinusoidal type functions as follows:

$Seas \left( t \right)=1+A_{s}\times cos(\frac{2\pi\left( t-\varphi_{s} \right)}{12})$ (Equation 5)

Here, amplitude $(A_{s})$ is a signal that swings above and below a reference value which was assumed to be 1. Phase angle ($\varphi_{s})$ is the time at which the peak is reached in a cycle.

We created the seasonal movement sub-model to overlay with the demographic sub-model to estimate the rates of movement among them. For example, rate of change in the transient population compartment ($T_{a}$) was calculated as a balance between seasonal movement, movement rates by age and region, aging, birth, and death, as follows:

$$\frac{dT_{a}}{dt}= seas.M_{a}^{NE\to T}.{NE}_{a}-seas.M_{a}^{T\to NE}.T_{a}-M^{T\to N}.T_{a}+birth-{death}_{a}$$

$-{aging}_{a}T_{a}+{migration}_{a}$ (Equation 6)

Rate of change in the Northeast population compartment (${NE}_{a}$), the group who live in the Northeast region for more than 6 months in a year, was calculated as a balance between, seasonal movement, movement rates by age and region, aging, birth, and death, as follows:

$\frac{d{NE}_{a}}{dt}= seas.M_{a}^{T\to NE}.T_{a}- seas.M_{a}^{NE\to T}.{NE}_{a}+M^{N\to NE}.{Non\_NE}_{a}- M^{NE\to N}.{NE}_{a}+birth-{death}_{a}-{aging}_{a}{NE}_{a}+{migration}_{a}$ (Equation 7)

Rate of change in the non-Northeast population compartment (${Non\_NE}_{a}$), the non-Northeast group, who live somewhere other than the Northeast, was calculated as a balance between movement rates by age and region, aging, birth, and death, as follows:

$\frac{d{Non\_NE}_{a}}{dt}= M^{T\to N}. T_{a}+M^{NE\to N}.{NE}_{a}-M^{N\to NE}.{Non\_NE}_{a}+birth-{death}_{a}-{aging}_{a}{Non\_NE}_{a}+{migration}_{a}$ (Equation 8)

All of the parameters included in the model are shown in S1 Table A (main text).

Table A Parameter table for the melioidosis infection model.

| **Parameter** | **Symbol** | **Value**  **(95% Credible Interval)** | **Source/**  **Reference** |
| --- | --- | --- | --- |
| **Population parameters** | | | |
| Fertility rate by age (per capita per month) | ${fr}_{a}$ |  | Census data [1] |
| Mortality rate by age (per capita per month) | ${dr}_{a}$ |  | Census data  [2] |
| Net international migration rate by age (per capita per month) | ${mr}_{a}$ |  | Thailand Migration Report  [3] |
| **Seasonal movement parameters** | | | |
| Amplitude | $A_{s}$ | 1 | Internal Migration in Thailand [4] |
| Phase angle | $\varphi_{s}$ | 6 | Internal Migration in Thailand [4] |
| Movement rate of Northeast to non-Northeast area (per capita per month) | $M^{NE\to N}$ | 0.136 (0.135-0.138) | Estimated |
| Movement rate of transient to non-Northeast area (per capita per month) | $M^{T\to N}$ | 0.076 (0.0745-0.077) | Estimated |
| Movement rate of non-Northeast to Northeast area (per capita per month) | $M^{N\to NE}$ | 0.070 (0.069-0.071) | Estimated |
| Movement rate of each age group among Northeast to transient area (per capita per month) | $M_{a}^{NE\to T}$ | Aged 0-14 = 0.00097 (0.00096-0.00099)  Aged 15-45 = 0.0041 (0.00406-0.00413)  Aged 46-60 = 0.0012 (0.0011-0.00113)  Aged 61-100 = 0.00004 (0.000037-0.000046) | Estimated |
| Movement rate of each age group among transient to Northeast area (per capita per month) | $M_{a}^{T\to NE}$ | Aged 0-14 = 0.04 (0.038-0.048)  Aged 15-45 = 0.12 (0.119-0.123)  Aged 46-60 = 0.19 (0.184-0.198)  Aged 61-100 = 0.005 (0.0001-0.01) | Estimated |
| **Diabetes parameters** | | | |
| Mortality/death rate for patients with diabetes (per capita per year) | ${dmr}_{a}$ | Aged 0-14 = 0.0009(0.0008-0.001)  Aged 15-39 =2.16(2.01-2.25)  Aged 40-49 =0.44(0.41-0.46)  Aged 50-59 =0.39(0.38-0.43)  Aged >= 60 =0.001(0.0005-0.005) | [5] |
| Diabetes incidence rate for each age group among females (per capita per year) | $K_{fa}^{DM}$ | Aged 0-39 = 0.001(0.0009-0.0012)  Aged 40-49 =0.011(0.01-0.012)  Aged 50-59 =0.032(0.031-0.033)  Aged >= 60 =0.026(0.025-0.027) | [5] |
| Diabetes incidence rate for each age group among males (per capita per year) | $K_{ma}^{DM}$ | Aged 0-39 = 0.005(0.004-0.006)  Aged 40-49 =0.017(0.016-0.018)  Aged 50-59 =0.021(0.02-0.022)  Aged >= 60 =0.018(0.017-0.019) | [5] |
| **Infection parameters** | | | |
| Infection rate (${10}^{-5})$ among males in the Northeast (per capita per month) | $\beta_{ma}^{NE}$ | Aged 0-14 = 0.7 (0.6-0.8)  Aged 15-44 = 0.7 (0.5-0.8)  Aged 45-59 = 6.1 (4.2-7.3)  Aged >= 60 = 1.7 (1.3-1.8) | Estimated |
| Infection rate (${10}^{-5})$ among females in the Northeast (per capita per month) | $\beta_{fa}^{NE}$ | Aged 0-14 = 0.5 (0.4-0.6)  Aged 15-44 = 0.3 (0.2-0.4)  Aged 45-59 = 2.8 (1.1-2.9)  Aged >= 60 = 0.8 (0.6-0.9) | Estimated |
| Infection rate (${10}^{-5})$ among males in the transient population (per capita per month) | $\beta_{ma}^{T}$ | Aged 0-14 = 0.3 (0.1-0.5)  Aged 15-44 = 0.8 (0.5-1.2)  Aged 45-59 = 0.8 (0.2-1.2)  Aged >= 60 = 1.1 (0.2-1.3) | Estimated |
| Infection rate (${10}^{-5})$ among females in the transient population (per capita per month) | $\beta_{fa}^{T}$ | Aged 0-14 = 1.6 (0.1-1.8)  Aged 15-44 = 0.3 (0.1-1.1)  Aged 45-59 = 0.3 (0.1-0.6)  Aged >= 60 = 0.7 (0.2-1.2) | Estimated |
| Infection rate (${10}^{-5})$ among males in the non-Northeast (per capita per month) | $\beta_{ma}^{N}$ | Aged 0-14 = 0.06 (0.05-0.08)  Aged 15-44 = 0.08 (0.04-0.09)  Aged 45-59 = 0.47 (0.4-0.65)  Aged >= 60 = 0.13 (0.12-0.2) | Estimated |
| Infection rate (${10}^{-5})$ among females in the non-Northeast (per capita per month) | $\beta_{fa}^{N}$ | Aged 0-14 = 0.09 (0.03-0.07)  Aged 15-44 = 0.04 (0.02-0.05)  Aged 45-59 = 0.19 (0.15-0.3)  Aged >= 60 = 0.08 (0.04-0.09) | Estimated |
| Incubation period (month) | $\nu$ | 0.5 | [6] |
| Proportion of symptomatic cases from exposer | $p^{E}$ | 0.46 (0.38-0.53) | Estimated |
| Recovery rate from asymptomatic (per capita per month) | $\sigma$ | 0.21 (0.005-0.24) | Estimated |
| Recovery rate from  symptomatic (per capita per month) | $\gamma$ | 0.11 (0.01-0.15) | Estimated |
| Relative susceptibility to melioidosis among diabetic individuals | $q$ | 10.84 (8.42 – 12.23) | Estimated |
| Recovery rate from  treatment (per capita per month) | $\rho$ | 2 | [7] |
| Severity rate from symptomatic (per capita per month) | $\theta$ | 3 | [8] |
| Treatment rate from symptomatic or severe cases (per capita per month) | $\tau$ | 3 | [7] |
| Treatment failure rate from severe cases (per capita per month) | $\delta$ | 5 | [9] |
| Loss of immunity rate among diabetic or non-diabetic patients  (per capita per month) | $\omega$ | 0.019 | [10] |
| Mortality/death rate for meliodosis patients (per capita per month) | $\mu_{M}$ | 0.6 (0.4-0.75) | Estimated |
| Proportion of reporting | *Report* | *Report* (in 2005-2009) = 0.17 (0.15-0.22)  *Report* (in 2010-2015) = 0.43 (0.39-0.54) | Estimated |
| **Seasonal parameters** | | | |
| Amplitude | $A_{inc}$ | 1.7 (1.3-1.8) | Estimated |
| Phase angle | $\varphi_{inc}$ | 20.6 (20.3-21.0) | Estimated |
| Pointiness of the curve | $pt$ | 2 | Assumed |

# Melioidosis infection sub-model

The melioidosis data for Thailand from 2008 to 2015 show seasonal patterns. The melioidosis incidence rate for each gender and age group*,* ${incML}_{ga}\left( t \right),$ has a sinusoidal type function as follows:

${incML}_{ga}\left( t \right)=\beta_{ga}\times{(1+A_{inc}\times cos(\pi(\frac{t-\varphi_{inc}}{12}))}^{pt})$ (Equation 9)

Here, infection rate ($\beta_{ga}$) is the probability or risk of an infection during a specific time period for each gender and age group between human and bacteria. Amplitude $(A_{inc})$ is a signal that swings above and below a reference value which was estimated as a peak amplitude. Phase angle ($\varphi_{inc})$ is the time at which the rate of infection is at its peak. $pt$ is the pointiness of the curve.

The diabetes incidence for each gender and age group (${incSDM}_{ga}$) [5] with gender, *g*, at age, *a*, at time, *t*, was taken to be a function of non-diabetic $S_{ga}$ with corresponding diabetes incidence rate $K_{ga}^{DM}$, which represents the rates of total diabetes, both diagnosed and undiagnosed, for each age group:

${incSDM}_{ga}(t)=K_{ga}^{DM}.S_{ga}(t)$ (Equation 10)

Number of deaths among diabetes (${deathSDM}_{ga}$) [5, 11] among both diagnosed and undiagnosed individuals in each age group, were a sum of the deaths from natural causes ${(dr}_{a})$ and the deaths resulting from diabetes itself, with is the mortality/death rate for diabetes, ${dmr}_{a}$*:*

${deathSDM}_{ga}\left( t \right)={(dr}_{a}+{dmr}_{a}).{SDM}_{ga}(t)$ (Equation 11)

Rates of change in ${NE}_{ga}(t)$, $T_{ga}(t)$, ${Non\_NE}_{ga}(t)$ were further divided into eight health compartments: susceptible (*S*), diabetic susceptible (*SDM*), exposed (*E*), symptomatic (*Sym*), asymptomatic (*Asym*), severe (*Sev*), treatment (*Treat*), and recovery (*R*), with the gender *g* and age group *a* represented by ordinary differential equations. For example, the rate of change of the susceptible compartment ($S_{ga}^{NE}$), a population at risk of melioidosis and free from diabetes, in Northeast to transient gender, *g*, at age, *a*, was represented by the following equation which describes the balance between birth inflows, diabetes incidence, melioidosis incidence, seasonal movement, aging, and death, as follows:

$\frac{dS_{ga}^{NE}}{dt}=seas.M_{a}^{T\to NE}.S_{ga}^{T}- seas.M_{a}^{NE\to T}.S_{ga}^{NE}+M^{N\to NE}.S_{ga}^{Non\_NE}-M^{NE\to N}.S_{ga}^{NE}+birth-{incML}_{ga}^{NE}.S_{ga}^{NE}+\omega.\left( 1-propDM \right).R_{ga}^{NE}-{incSDM}_{ga}-{death}_{ga}-{aging}_{a}S_{ga}^{NE}+{migration}_{ga}$ (Equation 12)

Rate of change in the diabetic susceptible compartment (${SDM}_{ga}^{NE}$), the population at risk of melioidosis who also had diabetes, was calculated as a balance between diabetes incidence, seasonal movement, aging, and death, as follows:

$\frac{d{SDM}_{ga}^{NE}}{dt}=seas.M_{a}^{T\to NE}.{SDM}_{ga}^{T}- seas.M_{a}^{NE\to T}.{SDM}_{ga}^{NE}+M^{NE\to N}.{SDM}_{ga}^{Non\_NE}-M_{a}^{NE\to N}.{SDM}_{ga}^{NE}-q.{incML}_{ga}^{NE}.{SDM}_{ga}^{NE}+\omega.propDM.R_{ga}^{NE}+{incSDM}_{ga}-d{eathSDM}_{ga}-{death}_{ga}-{aging}_{a}{SDM}_{ga}^{NE}+{migration}_{ga}$ (Equation 13)

Rate of change in the exposed compartment ($E_{ga}^{NE}$), the population who were exposed to melioidosis, was calculated as a balance between seasonal movement, infection rate, aging, and death, as follows:

$\frac{dE_{ga}^{NE}}{dt}=seas.M_{a}^{T\to NE}.E_{ga}^{T}- seas.M_{a}^{NE\to T}.E_{ga}^{NE}+M^{N\to NE}.E_{ga}^{Non\_NE}-M^{NE\to N}.E_{ga}^{NE}+{incML}_{ga}^{NE}.S_{ga}^{NE}+q.{incML}_{ga}^{NE}.{SDM}_{ga}^{NE}-\nu.E_{ga}^{NE}-d{eath}_{ga}-{aging}_{a}E_{ga}^{NE}+{migration}_{ga}$ (Equation 14)

Rate of change in the symptomatic compartment (${Sym}_{ga}^{NE}$), i.e. symptomatic melioidosis patients, was calculated as a balance between seasonal movement, incubation period, aging, and death, as follows:

$\frac{d{Sym}_{ga}^{NE}}{dt}=seas.M_{a}^{T\to NE}.{Sym}_{ga}^{T}- seas.M_{a}^{NE\to T}.{Sym}_{ga}^{NE}+M^{N\to NE}.{Sym}_{ga}^{Non\_NE}-M^{NE\to N}.{Sym}_{ga}^{NE}+p^{E}.\nu.E_{ga}^{NE}-\gamma.{Sym}_{ga}^{NE}-\theta.{Sym}_{ga}^{NE}-\tau.{Sym}_{ga}^{NE}-d{eath}_{ga}-{aging}_{a}{Sym}_{ga}^{NE}+{migration}_{ga}$ (Equation 15)

Rate of change in the asymptomatic compartment (${Asym}_{ga}^{NE}$), i.e. asymptomatic melioidosis patients, was calculated as a balance between seasonal movement, incubation period, aging, and death, as follows:

$\frac{d{Asym}_{ga}^{NE}}{dt}=seas.M_{a}^{T\to NE}.{Asym}_{ga}^{T}- seas.M_{a}^{NE\to T}.{Asym}_{ga}^{NE}+M^{N\to NE}.{Asym}_{ga}^{Non\_NE}-M^{NE\to N}.{Asym}_{ga}^{NE}+\left( 1-p^{E} \right).\nu.E_{ga}^{NE}-\sigma.{Asym}_{ga}^{NE}-d{eath}_{ga}-{aging}_{a}{Asym}_{ga}^{NE}+{migration}_{ga}$ (Equation 16)

Rate of change in the severe compartment (${Sev}_{ga}^{NE}$), i.e. melioidosis patients with severe symptoms, was calculated as a balance between seasonal movement, severity rate, aging, and death, as follows:

$\frac{d{Sev}_{ga}^{NE}}{dt}=seas.M_{a}^{T\to NE}.{Sev}_{ga}^{T}- seas.M_{a}^{NE\to T}.{Sev}_{ga}^{NE}+M^{N\to NE}.{Sev}_{ga}^{Non\_NE}-M^{NE\to N}.{Sev}_{ga}^{NE}+\theta.{Sym}_{ga}^{NE}-\tau.{Sev}_{ga}^{NE}+\delta.{Treat}_{ga}^{NE}-\mu_{M}.{Sev}_{ga}^{NE}-d{eath}_{ga}-{aging}_{a}{Sev}_{ga}^{NE}+{migration}_{ga}$ (Equation 17)

Rate of change in the treatment compartment (${Treat}_{ga}^{NE}$), i.e. melioidosis patients who received treatment, was calculated as a balance between seasonal movement, treatment rate, aging, and death, as follows:

$\frac{d{Treat}_{ga}^{NE}}{dt}=seas.M_{a}^{T\to NE}.{Treat}_{ga}^{T}- seas.M_{a}^{NE\to T}.{Treat}_{ga}^{NE}+M^{N\to NE}.{Treat}_{ga}^{Non\_NE}-M^{NE\to N}.{Treat}_{ga}^{NE}+\theta.{Sym}_{ga}^{NE}-\rho.{Treat}_{ga}^{NE}+\tau.{Sym}_{ga}^{NE}-\tau.{Sev}_{ga}^{NE}-d{eath}_{ga}-{aging}_{a}{Treat}_{ga}^{NE}+{migration}_{ga}$ (Equation 18)

Rate of change in the recovered compartment ($R_{ga}^{NE}$), i.e. melioidosis patients who had recovered, was calculated as a balance between seasonal movement, recovery rate, aging, and death, as follows:

$\frac{dR_{ga}^{NE}}{dt}=seas.M_{a}^{T\to NE}.R_{ga}^{T}- seas.M_{a}^{NE\to T}.R_{ga}^{NE}+M^{N\to NE}.R_{ga}^{Non\_NE}-M^{NE\to N}.R_{ga}^{NE}+\gamma.{Sym}_{ga}^{NE}+\sigma.{Asym}_{ga}^{NE}-\omega.R_{m1}^{NE}+\rho.{Treat}_{ga}^{NE}-d{eath}_{ga}-{aging}_{a}R_{ga}^{NE}+{migration}_{ga}$ (Equation 19)

Cumulative mortality (${CumMor}_{ga})$ was analyzed by numerical integration of the corresponding severe cases (${Sev}_{ga}^{NE})$ and mortality rates among severe cases ($\mu_{M}$), calculated as follows:

${CumMor}_{ga}=\int_{t}^{t+1} \mu_{M}.{Sev}_{ga}^{NE}dt$ (Equation 20)

Cumulative incidence (${CumInc}_{ga})$ was analyzed by numerical integration of the corresponding susceptible cases ($S_{ga}^{NE})$, diabetic susceptible cases (${SDM}_{ga}^{NE}$), and incidence rates for each age group (${incML}_{ga}^{NE}$), calculated as follows:

${CumInc}_{ga}=\int_{t}^{t+1} \left( {incML}_{ga}^{NE}.S_{ga}^{NE}+q.{incML}_{ga}^{NE}.{SDM}_{ga}^{NE} \right) dt$ (Equation 21)

Cumulative hospitalized cases (${CumHos}_{ga})$ was analyzed by numerical integration of the corresponding symptomatic cases (${Sym}_{ga}^{NE})$, severe cases (${Sev}_{ga}^{NE}$), and treatment rates ($\tau$), multiplied by reporting proportion (*report*) and calculated as follows:

$Cum{Hos}_{ga}=\int_{t}^{t+1} ({\tau.Sym}_{ga}^{NE}+\tau.{Sev}_{ga}^{NE}).report dt$

(Equation 22)

Other reported measures were treated similarly.

# References

1. MoPH. Public health statistics A.D.2010. Thailand: Ministry of Public health; 2010.

2. MoPH. Public health statistics A.D.2000. Thailand: Ministry of Public health; 2000.

3. Huguet JW. Thailand Migration Report 2011. Thailand: International Organization for Migration, 2011.

4. Guest P, Chamratrithirong A, Archavanitkul K, Piriyathamwong N, Richter K. Internal migration in Thailand. Asian Pac Migr J. 1994;3(4):531-45. Epub 1994/01/01. PubMed PMID: 12346388.

5. Mahikul W, White, L., Poovorawan, K., Soonthornworasiri, N., Sukontamarn, P., Chanthavilay, P., Pan-ngum, W., Medley, G. A Population Dynamic Model to Assess the Diabetes Screening and Reporting Programs and Project the Burden of Undiagnosed Diabetes in Thailand. In: University M, editor. 2018.

6. Currie BJ. Burkholderia pseudomallei and Burkholderia mallei: Melioidosis and Glanders. Australia: Bentham Science Publishers; 2007.

7. White NJ. Melioidosis. Lancet. 2003;361(9370):1715-22. PubMed PMID: 12767750.

8. Limmathurotsakul D, Peacock SJ. Melioidosis: a clinical overview. Br Med Bull. 2011;99:125-39. doi: 10.1093/bmb/ldr007. PubMed PMID: 21558159.

9. Simpson AJ, Suputtamongkol Y, Smith MD, Angus BJ, Rajanuwong A, Wuthiekanun V, et al. Comparison of imipenem and ceftazidime as therapy for severe melioidosis. Clin Infect Dis. 1999;29(2):381-7. Epub 1999/09/07. doi: 10.1086/520219. PubMed PMID: 10476746.

10. Limmathurotsakul D, Chaowagul W, Chierakul W, Stepniewska K, Maharjan B, Wuthiekanun V, et al. Risk factors for recurrent melioidosis in northeast Thailand. Clin Infect Dis. 2006;43(8):979-86. Epub 2006/09/20. doi: 10.1086/507632. PubMed PMID: 16983608.

11. Pratipanawatr T, Rawdaree P, Chetthakul T, Bunnag P, Ngarmukos C, Benjasuratwong Y, et al. Thailand Diabetic Registry cohort: predicting death in Thai diabetic patients and causes of death. J Med Assoc Thai. 2010;93 Suppl 3:S12-20. PubMed PMID: 21299087.
