# Supplementary material for: Modelling population dynamics and seasonal movement to assess and predict the burden of melioidosis
Source: PLoS Negl Trop Dis. 2019 May 9;13(5):e0007380. doi: 10.1371/journal.pntd.0007380 (PMC6529009; doi:10.1371/journal.pntd.0007380)
Supplement: S2 File — Figure (A). Projection of the population size of Thailand between 1980 and 2035. Figure (B). Observed data and model estimates of the annual population (in millions) in Northeast and non-Northeast Thailand during 2005–2010. Figure (C). Estimation of the transient population by age (in thousands) in Thailand, from 2005 to 2015. Figure (D). Posterior distributions from the melioidosis infection model, that each row corresponds to the separate parameter, the left-hand column contains traces with 6 color chains and the right-hand column contains the posterior distribution, corresponding to each parameter.Table (A). Estimation of the number of deaths of males and females from melioidosis by age group for selected years. Bayesian framework. Bayes theorem, Prior distribution, Likelihood function and Posterior estimation. (DOCX) [file pntd.0007380.s002.docx]

**Supporting Information (S2)**

## The Bayesian framework

Bayesian inference of seasonal movement and a melioidosis infection model provide a framework for estimating parametric uncertainty in terms of probabilistic distributions and allowing a direct quantification of parameter uncertainty.

Bayes theorem states that the best estimate (posterior uncertainty $p(\theta|y$)) for a parameter vector $\theta$ given data y is given by:

$p\left( \theta| y \right)=\frac{p\left( \theta\right)p(y|\theta)}{p(y)}$ (Equation 1)

Here, $p\left( \theta\right)$ is the prior information and*,*$\frac{p(y|\theta)}{p(y)}$ is the likelihood ratio. Markov Chain Monte Carlo *(MCMC)* algorithms were applied to approximate these distributions which used a sampling scheme to estimate the posterior distribution [1, 2].

## *Prior distribution*

Uniform distribution was chosen to be the prior distribution for all parameter values, given that little information about these parameters has been measured or reported. The minimum and maximum values were initially determined then narrowed down from the iterative model fitting procedure.

## *Likelihood function*

We defined the likelihood as the product of likelihood terms for each data point. The data arise from the averaged migrant data from 2005 to 2015 and the annual epidemiological surveillance report between 2005 and 2015 and are linked to the summation of expected age and gender rates via a Poisson distribution. The log-likelihood (used as the target in the MCMC algorithm) is:

Likelihood function of seasonal movement model

${LL}_{seas}=\sum_{a} \left( \sum_{t} log\left( \frac{{Ms}^{\theta s}exp(-Ms)}{\theta s!} \right) \right)$ (Equation 2)

Where *θs* is the number of transient population age *a* time *t* and *Ms* is the expected number of transient population from the model at each age class *a* and time *t*.

The likelihood function of melioidosis model:

${LL}_{incML}=\sum_{a} \left( \sum_{t} log\left( \frac{{Mm}^{\theta m}exp(-Mm)}{\theta m!} \right) \right)$ (Equation 3)

Where *θm* is the incidence of melioidosis at each age class *a* and time *t*, and *Mm* is the expected incidence of melioidosis by model at each age class *a* and time *t*.

## *Posterior estimation*

We used a Differential Evolution Markov Chain Monte Carlo (MCMCzs, or DE-MCzs) method to estimate the posterior distributions. We considered Markov Chain methods of sampling that were proposed by Ter Braak and Vrugt et al, 2008 [3], which have previously been used for numerical problems, and implemented using the Bayesian Tools R package. Differential Evolution Markov Chain (DE-MC) is an adaptive MCMC algorithm, in which multiple chains are run in parallel and presented. The DE-MCzs method combines characteristics of conventional MCMC methods with the ideas of differential evolution optimization algorithms, by making use of the full joint density function and (independent) proposal distributions for each of the variables including reporting, seasonal movement, and incidence rate of melioidosis. These samples are accepted probabilistically based on the acceptance probability. Uniform distributions are centered at the current state of the chain. This proposal distribution randomly perturbs the current state of the chain, and then either accepts or rejects the perturbed value. Two chains ran in parallel on three cores, each consisting of 100,000 iterations and a burn-in period of 25,000 iterations for seasonal movement model, were run in parallel to achieve a target acceptance rate of 0.2. While, melioidosis model was ran for 5,000 iterations, and a burn-in period of 1,000 iterations in parallel to achieve a target acceptance rate of 0.15. The display of the two-split chains when using the Bayesian package was 6 lines of (5,000-1,000)/3 = 1,333 shown in Figure D.


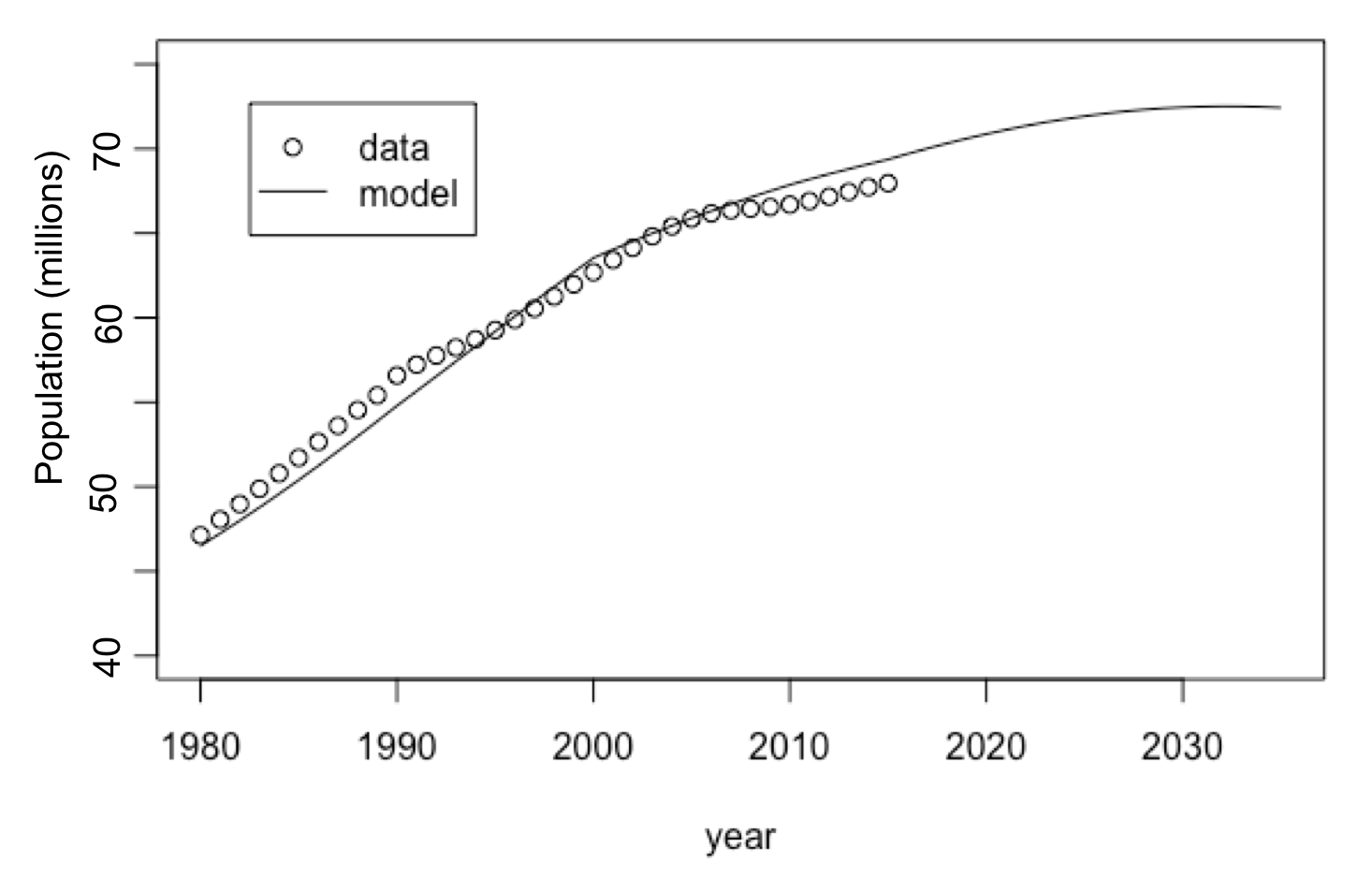


Figure A. Projection of the population size of Thailand between 1980 and 2035. White dot: total population each year; line: model.


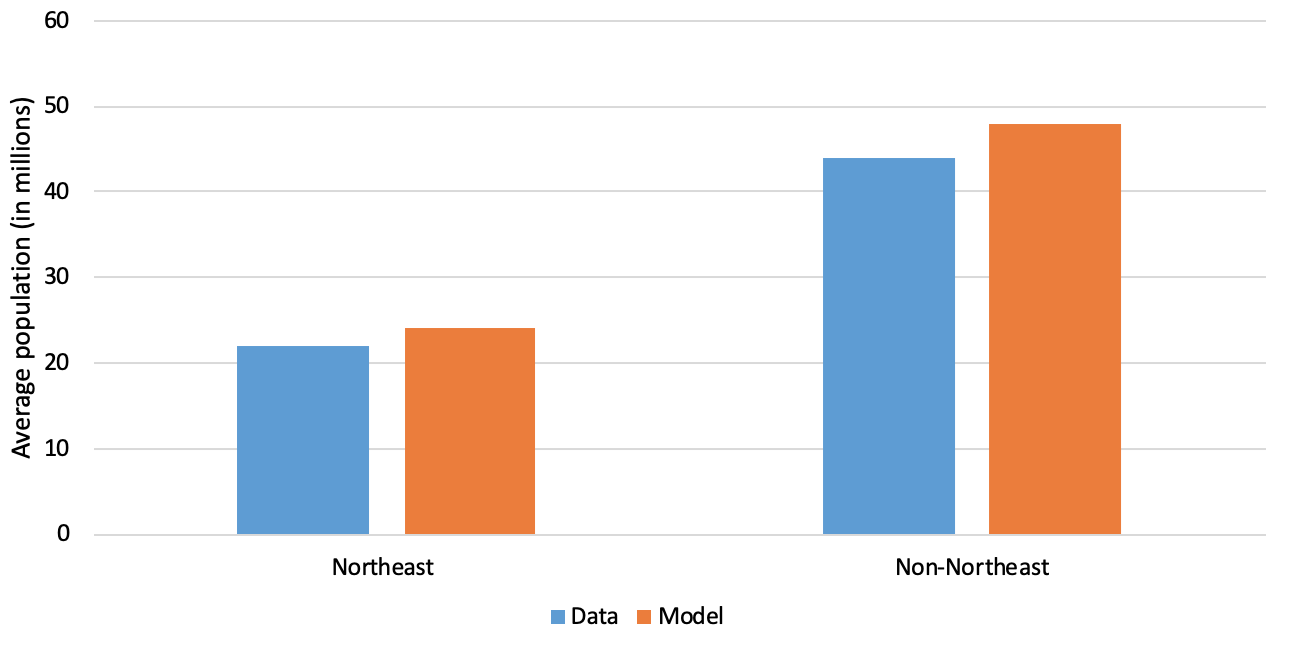


**Figure B. Observed data and model estimates of the average population per year (in millions) in Northeast and non-Northeast Thailand during 2005-2010.**


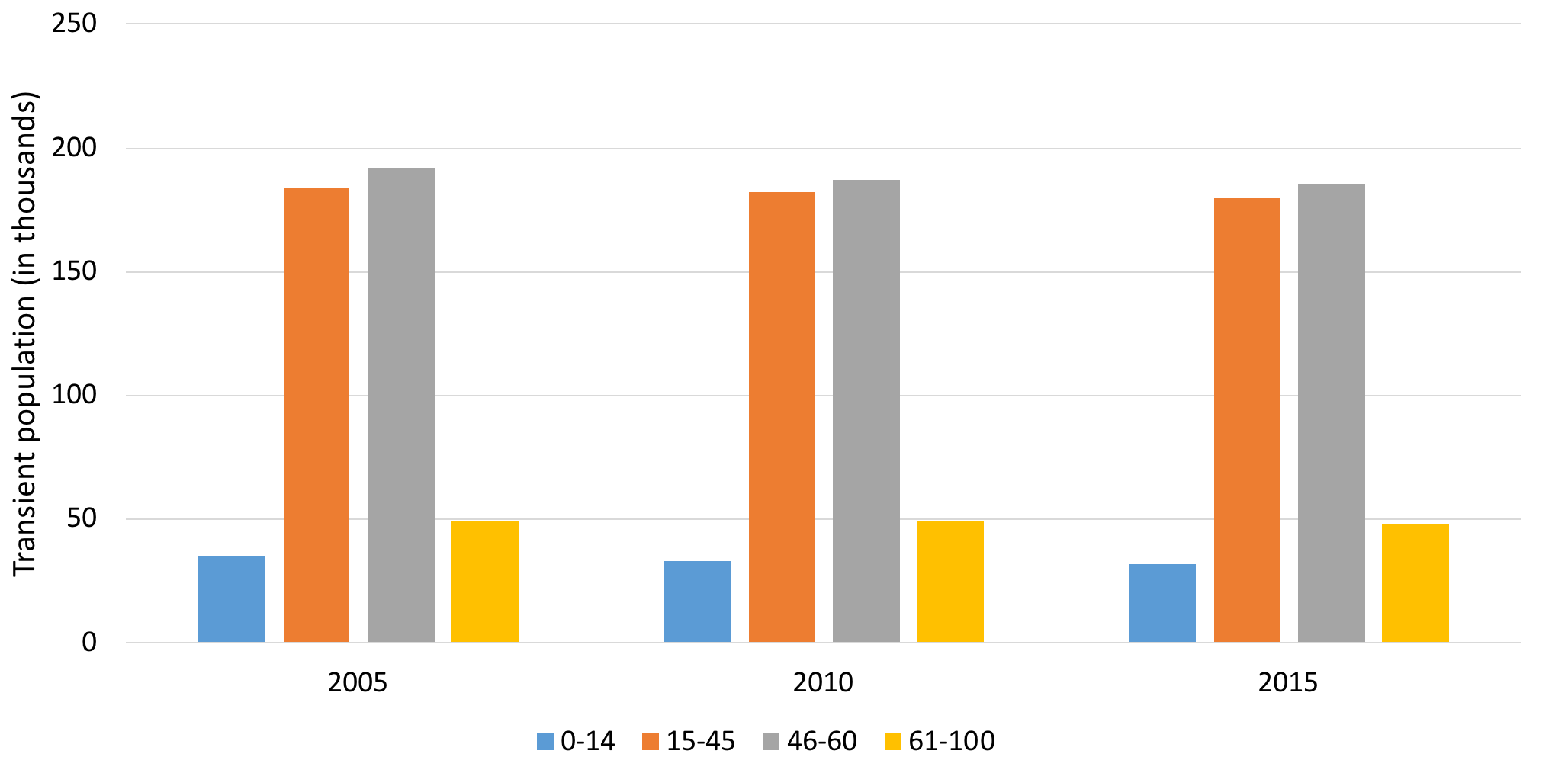


Figure C. Estimation of the transient population by age (in thousands) in Thailand, from 2005 to 2015.


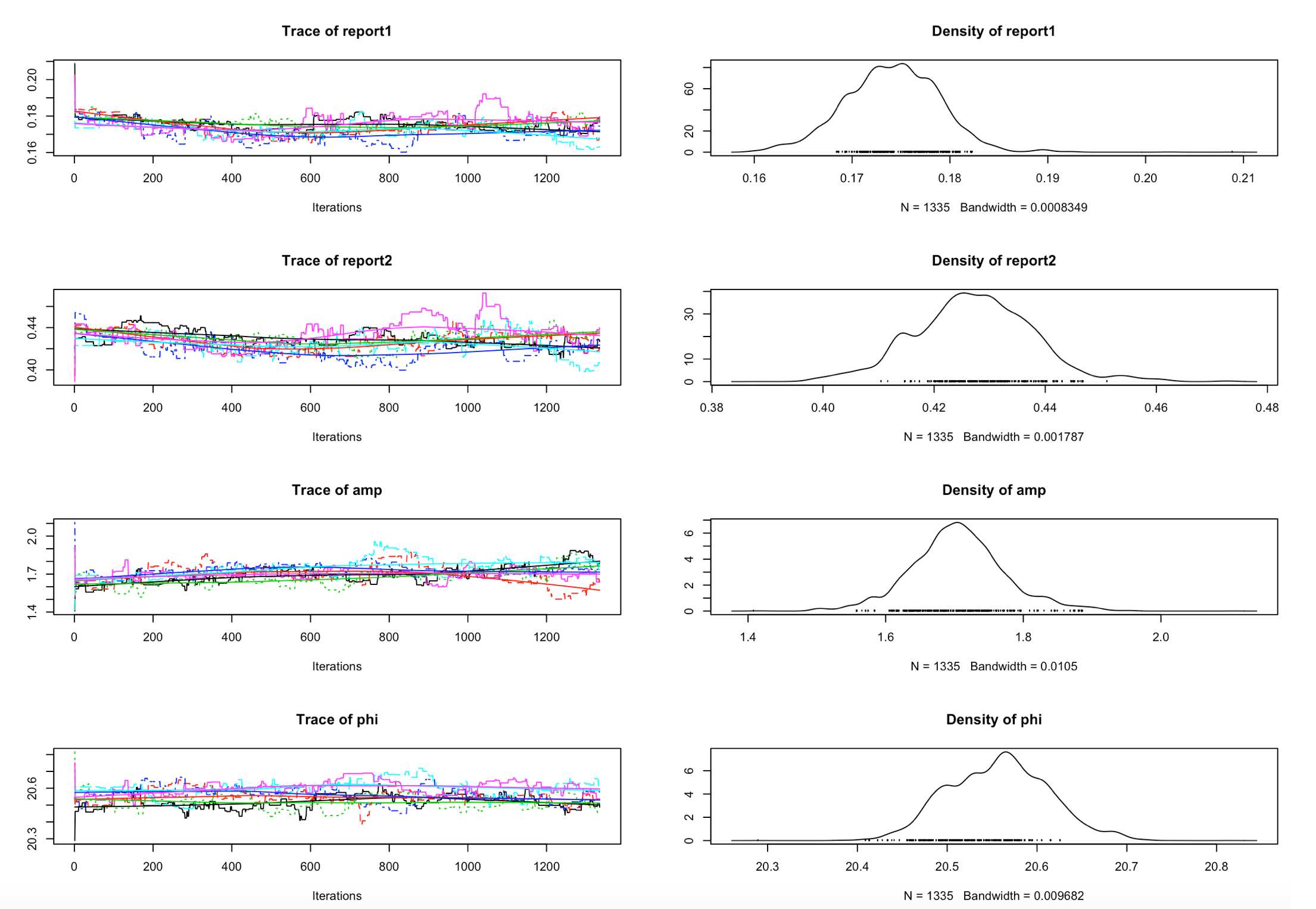

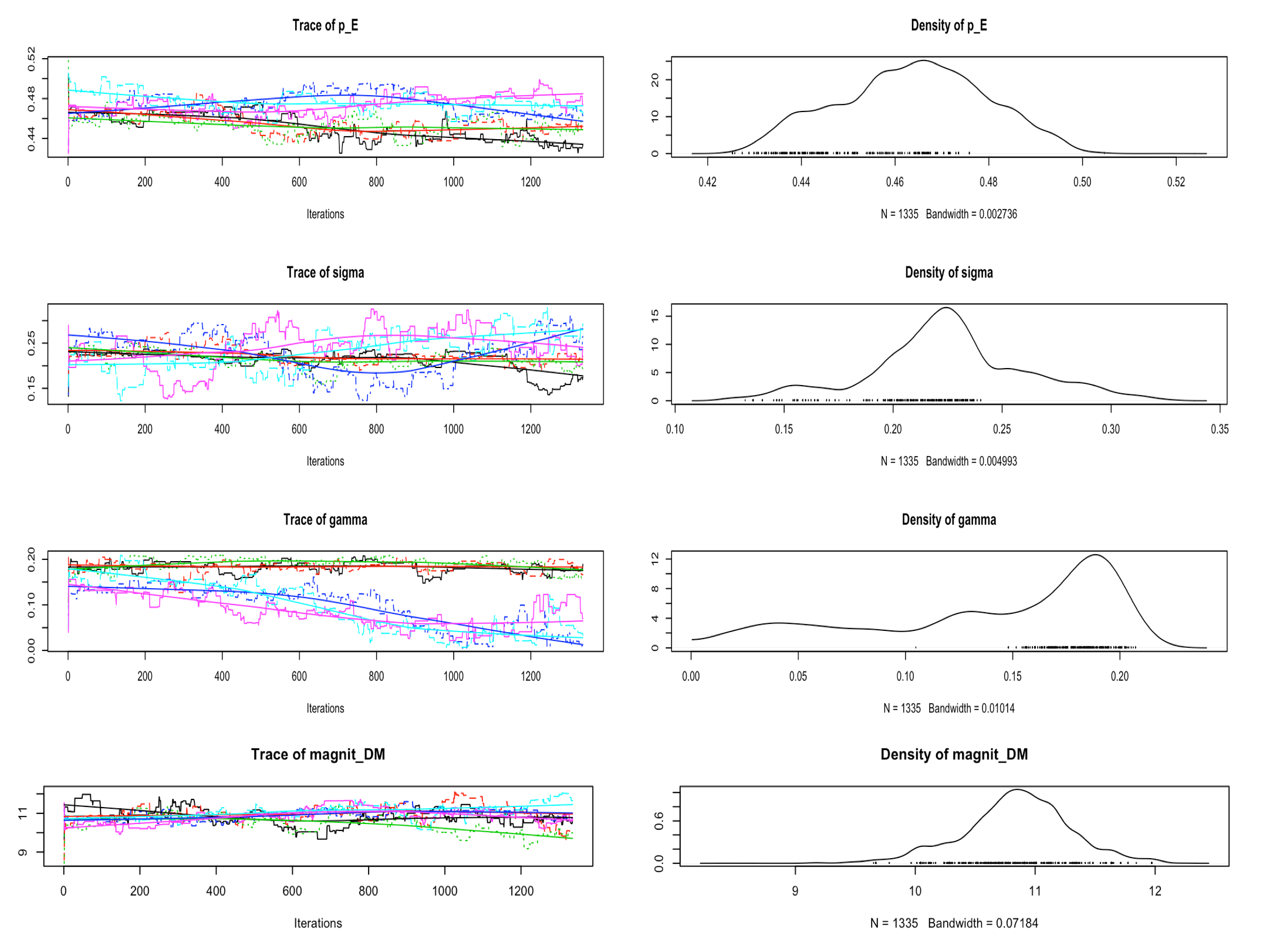

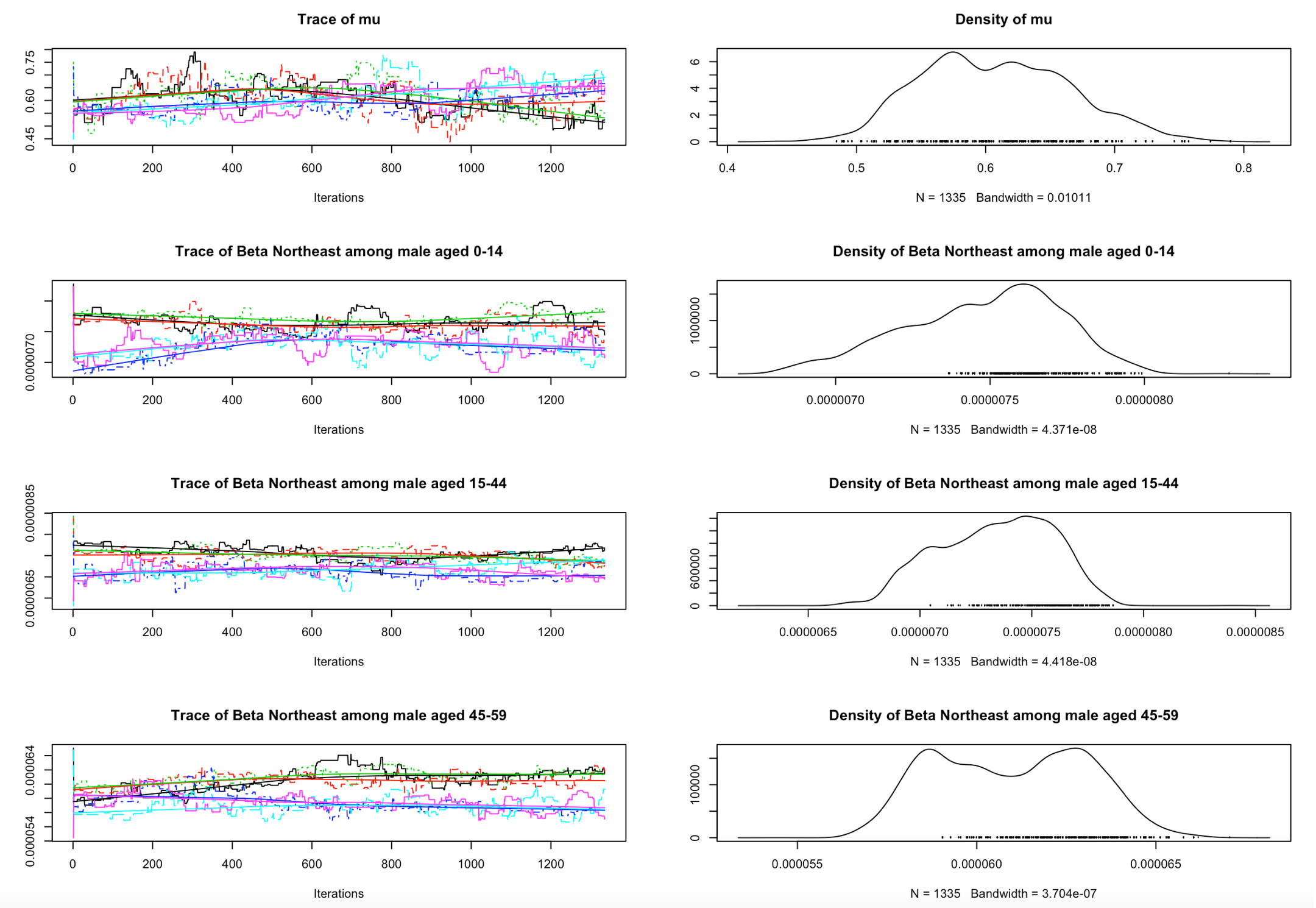

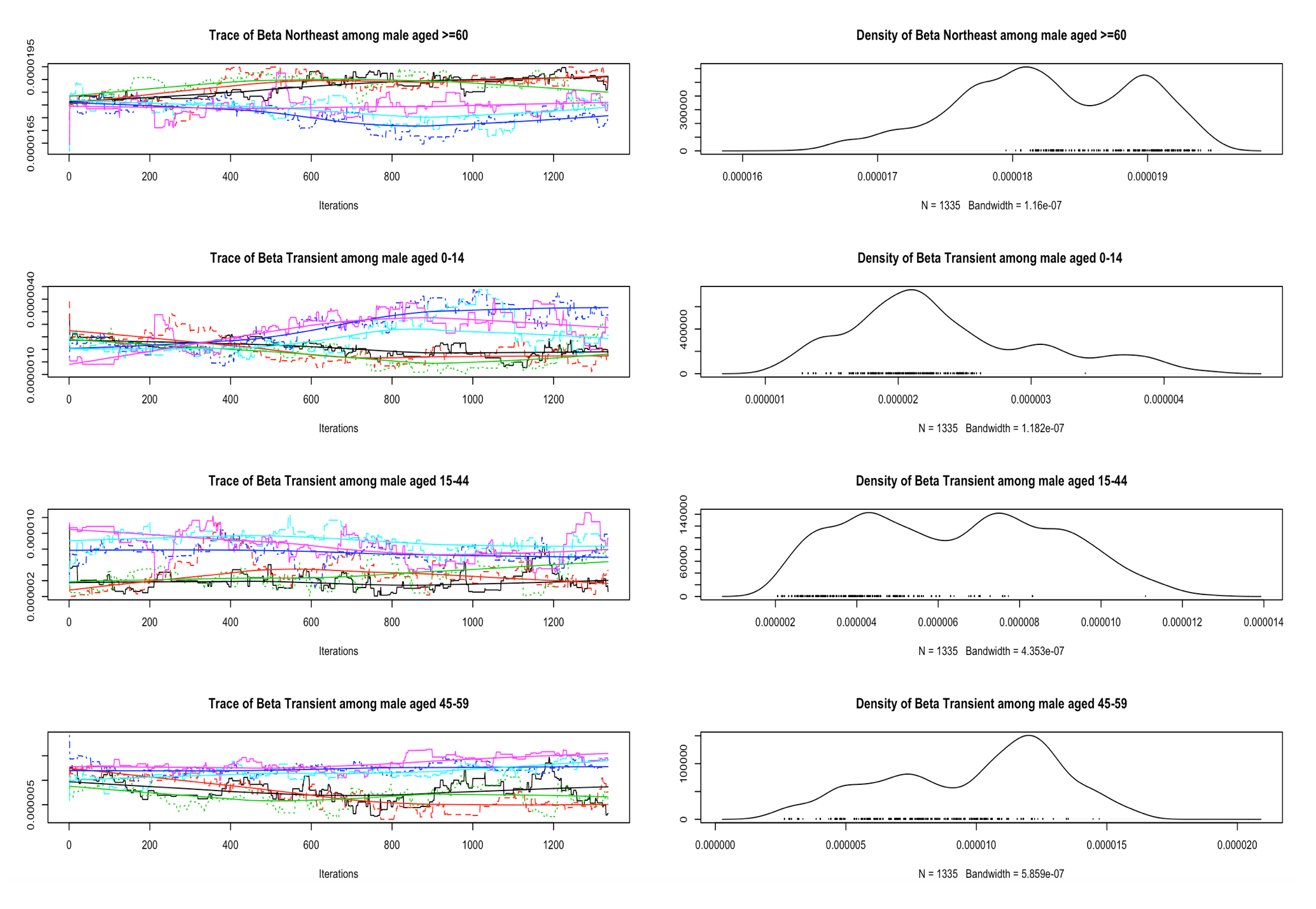

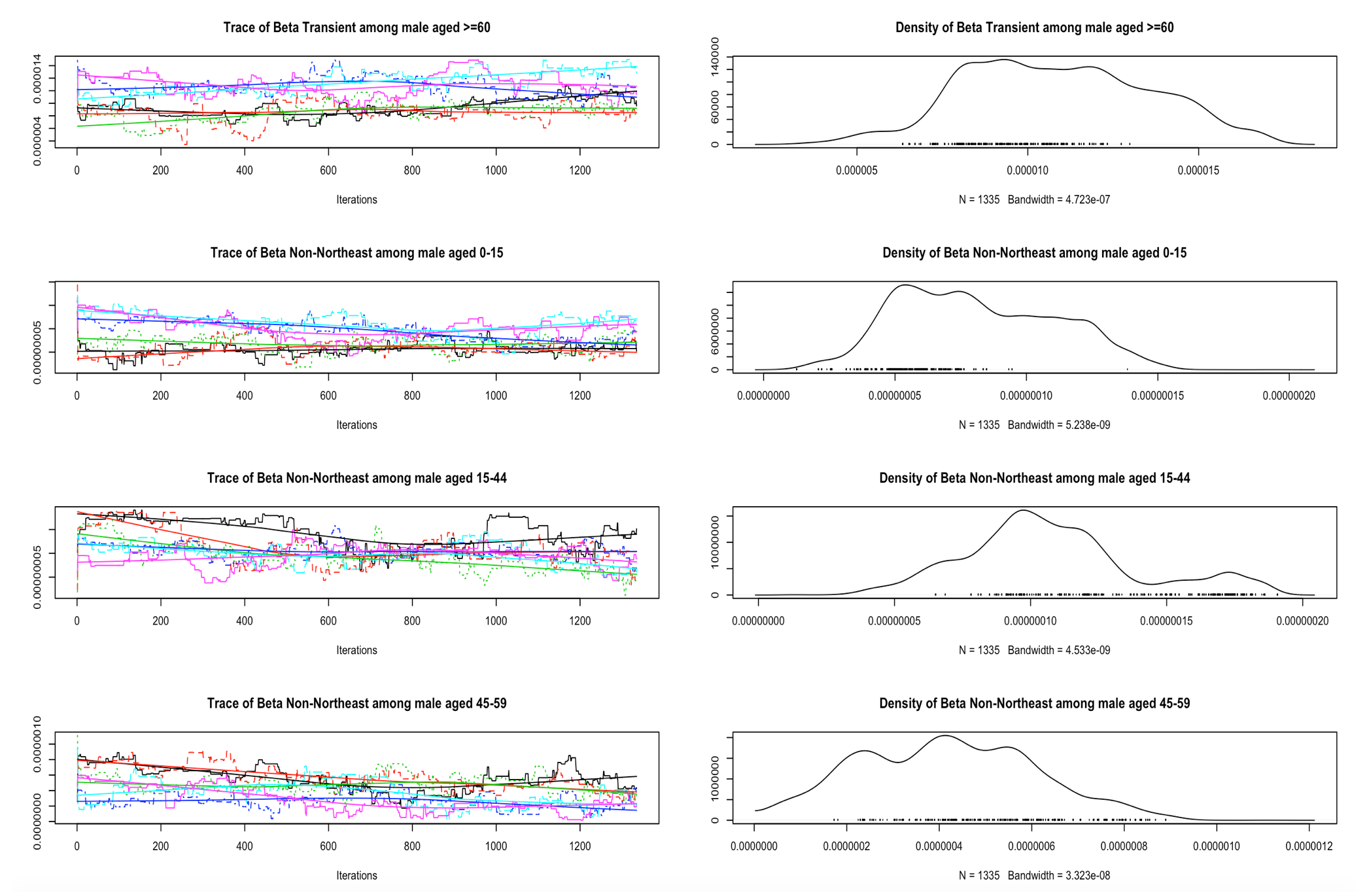

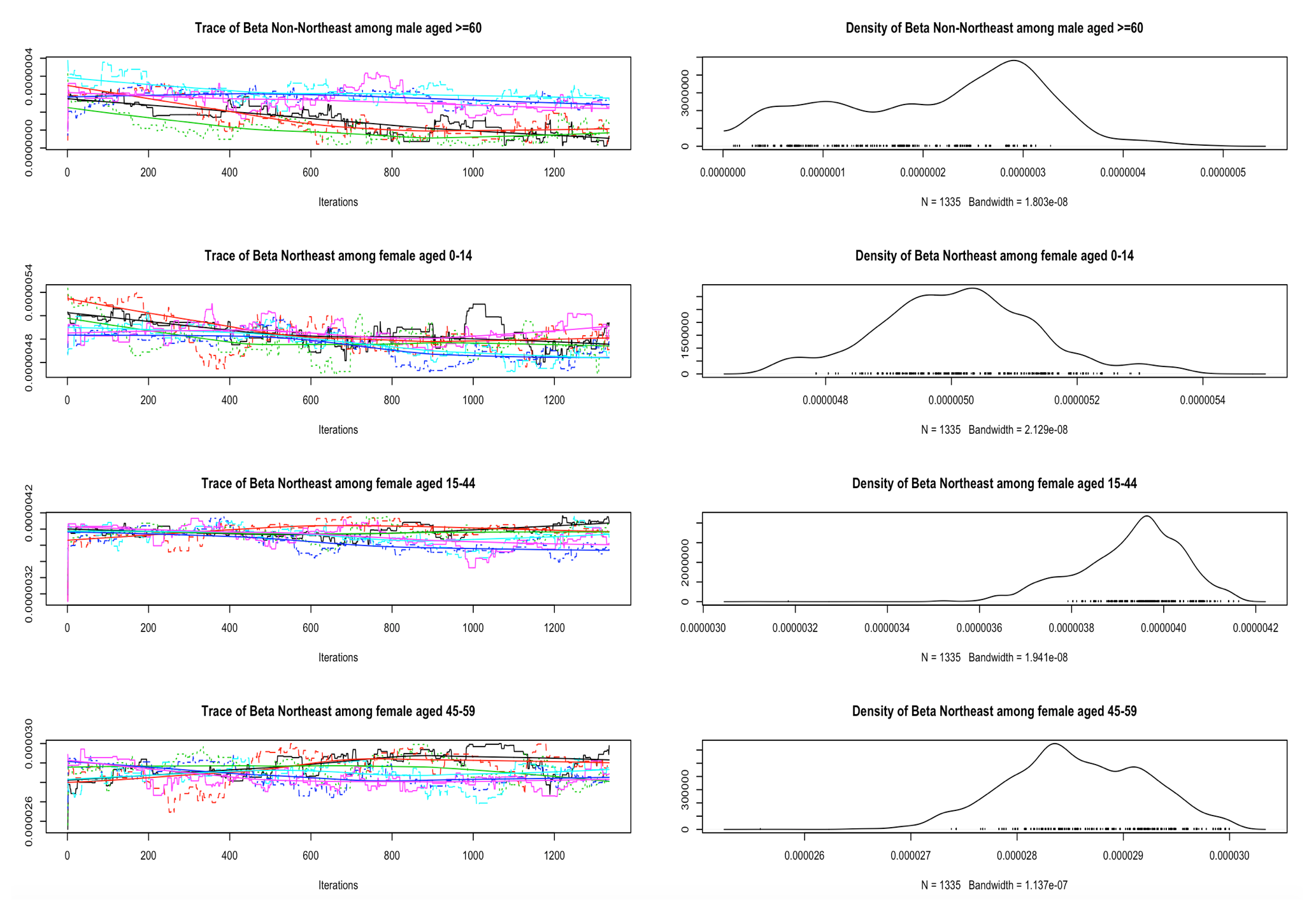

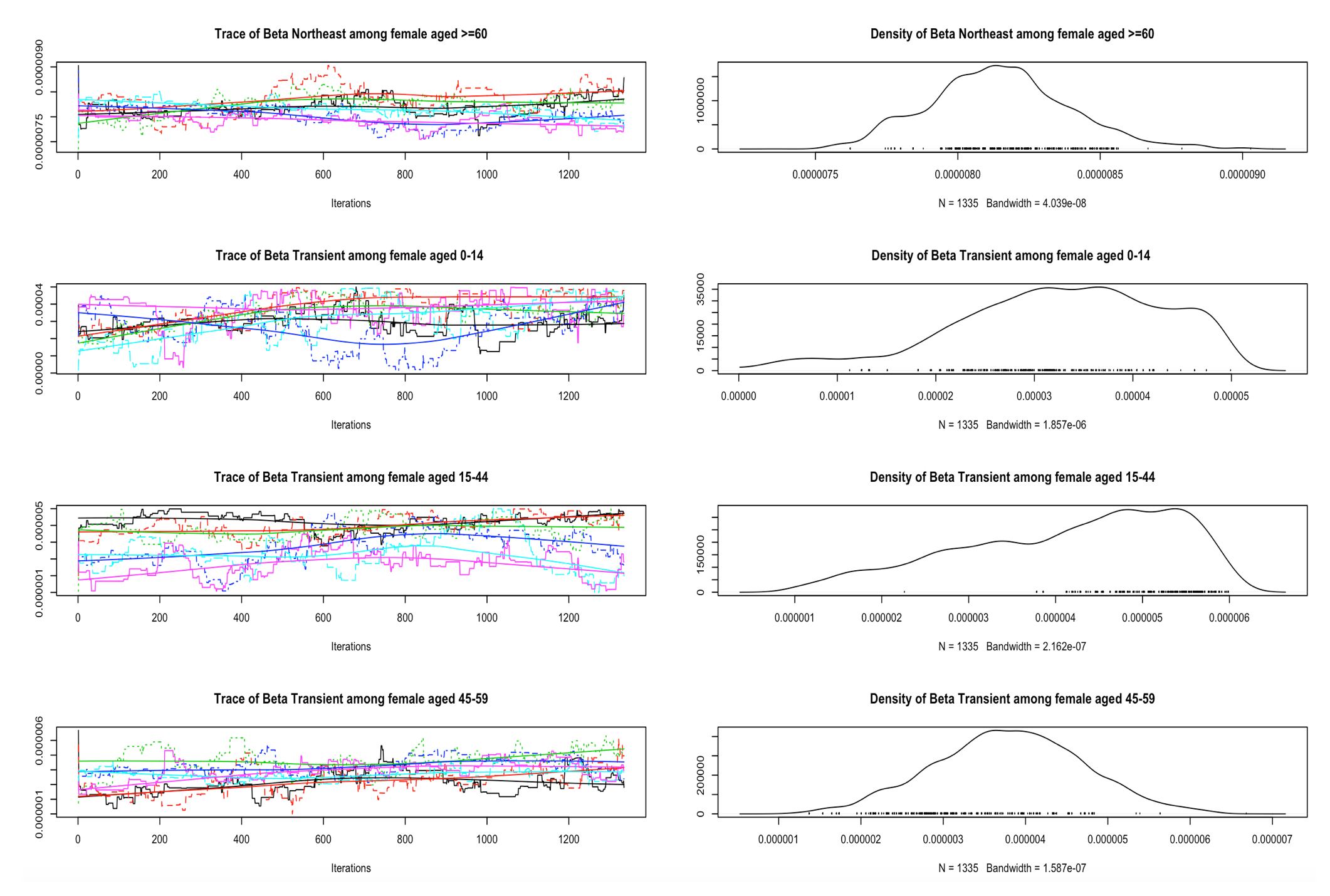

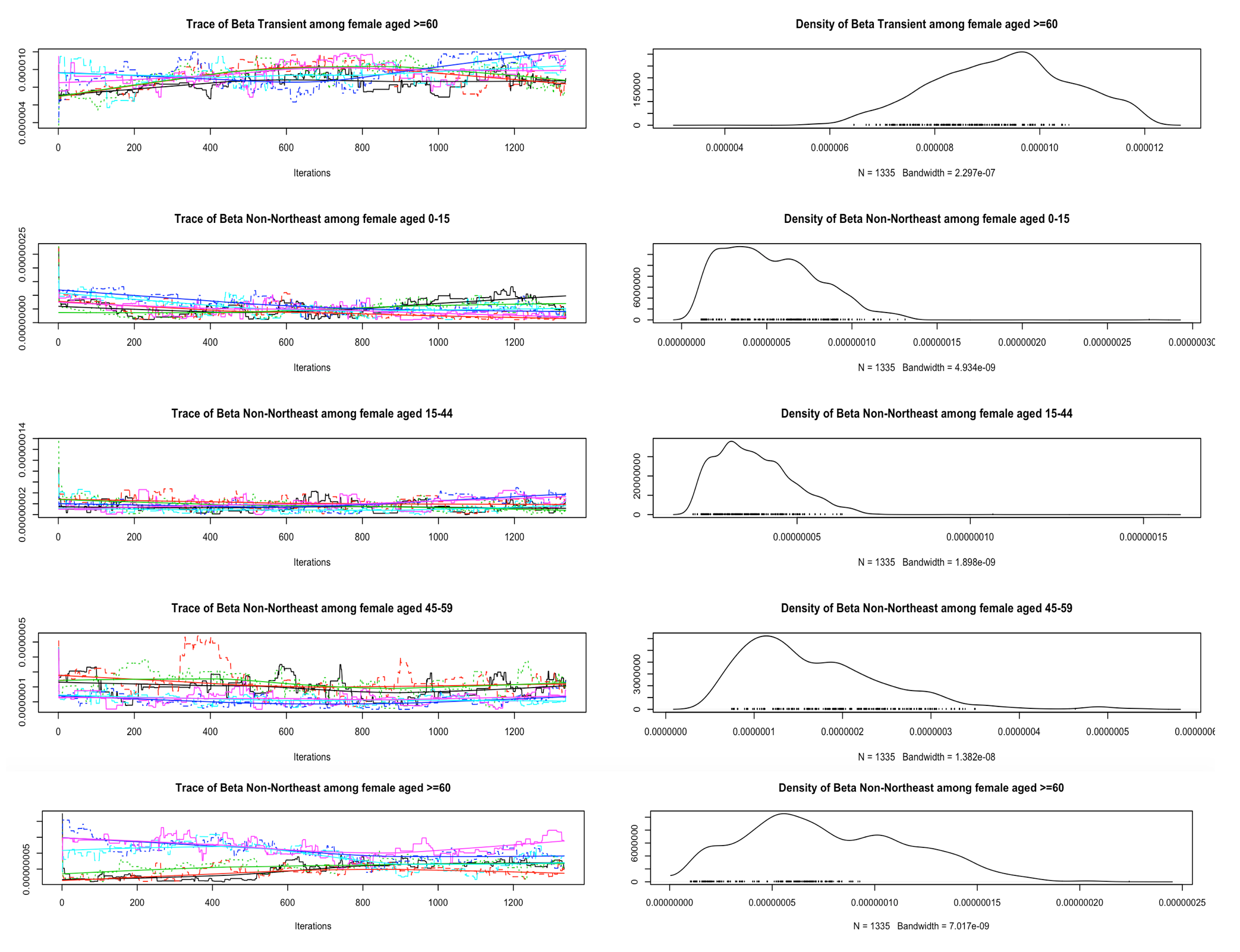


**Figure D. Posterior distributions from the melioidosis infection model, that each row corresponds to the separate parameter, the left-hand column contains traces with 6 color chains (dashed lines: actual traces, solid lines: trends) and the right-hand column contains the posterior distribution, corresponding to each parameter.**

Table A. Estimation of the number of deaths in males and females from melioidosis by age group for selected years.

| year | Age-group (years) | | | | | | | | Total |
| --- | --- | --- | --- | --- | --- | --- | --- | --- | --- |
|  | 0-14 | | 15-44 | | 45-59 | | ≥60 | |  |
|  | Male | Female | Male | Female | Male | Female | Male | Female |  |
| 2005 | 126 | 77 | 108 | 66 | 262 | 162 | 164 | 104 | 1,069 |
| 2010 | 141 | 86 | 136 | 85 | 318 | 202 | 191 | 125 | 1,284 |
| 2015 | 144 | 89 | 152 | 95 | 359 | 232 | 203 | 139 | 1,413 |
| 2035 | 177 | 109 | 186 | 115 | 557 | 362 | 288 | 215 | 2,009 |

## References

1. Augustynczik ALD, Hartig F, Minunno F, Kahle H-P, Diaconu D, Hanewinkel M, et al. Productivity of Fagus sylvatica under climate change – A Bayesian analysis of risk and uncertainty using the model 3-PG. Forest Ecology and Management. 2017;401(Supplement C):192-206. doi: <https://doi.org/10.1016/j.foreco.2017.06.061>.

2. Hartig F, Minunno, F., Paul, S.,. BayesianTools: General-Purpose MCMC and SMC Samplers and Tools for Bayesian Statistics. R package version. R package version 0.1.3. 2017. Available from: <https://cran.r-project.org/web/packages/BayesianTools/ndex.html>.

3. Ter Braak CJ, Vrugt, J.A. Differential evolution Markov chain with snooker updater and fewer chains. Stat Comput 2008;18:435–46.
